# Telomere-to-telomere carrot (*Daucus carota*) genome assembly reveals carotenoid characteristics

**DOI:** 10.1093/hr/uhad103

**Published:** 2023-05-10

**Authors:** Ya-Hui Wang, Pei-Zhuo Liu, Hui Liu, Rong-Rong Zhang, Yi Liang, Zhi-Sheng Xu, Xiao-Jie Li, Qing Luo, Guo-Fei Tan, Guang-Long Wang, Ai-Sheng Xiong

**Affiliations:** State Key Laboratory of Crop Genetics & Germplasm Enhancement and Utilization, Ministry of Agriculture and Rural Affairs Key Laboratory of Biology and Germplasm Enhancement of Horticultural Crops in East China, College of Horticulture, Nanjing Agricultural University, Nanjing, Jiangsu 210095, China; State Key Laboratory of Crop Genetics & Germplasm Enhancement and Utilization, Ministry of Agriculture and Rural Affairs Key Laboratory of Biology and Germplasm Enhancement of Horticultural Crops in East China, College of Horticulture, Nanjing Agricultural University, Nanjing, Jiangsu 210095, China; State Key Laboratory of Crop Genetics & Germplasm Enhancement and Utilization, Ministry of Agriculture and Rural Affairs Key Laboratory of Biology and Germplasm Enhancement of Horticultural Crops in East China, College of Horticulture, Nanjing Agricultural University, Nanjing, Jiangsu 210095, China; State Key Laboratory of Crop Genetics & Germplasm Enhancement and Utilization, Ministry of Agriculture and Rural Affairs Key Laboratory of Biology and Germplasm Enhancement of Horticultural Crops in East China, College of Horticulture, Nanjing Agricultural University, Nanjing, Jiangsu 210095, China; Beijing Vegetable Research Center, Beijing Academy of Agriculture and Forestry Sciences, Key Laboratory of Biology and Genetic Improvement of Horticultural Crops in North China, Beijing 100097, China; State Key Laboratory of Crop Genetics & Germplasm Enhancement and Utilization, Ministry of Agriculture and Rural Affairs Key Laboratory of Biology and Germplasm Enhancement of Horticultural Crops in East China, College of Horticulture, Nanjing Agricultural University, Nanjing, Jiangsu 210095, China; Beijing Vegetable Research Center, Beijing Academy of Agriculture and Forestry Sciences, Key Laboratory of Biology and Genetic Improvement of Horticultural Crops in North China, Beijing 100097, China; Institute of Horticulture, Guizhou Academy of Agricultural Sciences, Guiyang, Guizhou 550025, China; Institute of Horticulture, Guizhou Academy of Agricultural Sciences, Guiyang, Guizhou 550025, China; School of Life Science and Food Engineering, Huaiyin Institute of Technology, Huaian, Jiangsu 223003, China; State Key Laboratory of Crop Genetics & Germplasm Enhancement and Utilization, Ministry of Agriculture and Rural Affairs Key Laboratory of Biology and Germplasm Enhancement of Horticultural Crops in East China, College of Horticulture, Nanjing Agricultural University, Nanjing, Jiangsu 210095, China

## Abstract

Carrot (*Daucus carota*) is an Apiaceae plant with multi-colored fleshy roots that provides a model system for carotenoid research. In this study, we assembled a 430.40 Mb high-quality gapless genome to the telomere-to-telomere (T2T) level of “Kurodagosun” carrot. In total, 36 268 genes were identified and 34 961 of them were functionally annotated. The proportion of repeat sequences in the genome was 55.3%, mainly long terminal repeats. Depending on the coverage of the repeats, 14 telomeres and 9 centromeric regions on the chromosomes were predicted. A phylogenetic analysis showed that carrots evolved early in the family Apiaceae. Based on the T2T genome, we reconstructed the carotenoid metabolic pathway and identified the structural genes that regulate carotenoid biosynthesis. Among the 65 genes that were screened, 9 were newly identified. Additionally, some gene sequences overlapped with transposons, suggesting replication and functional differentiation of carotenoid-related genes during carrot evolution. Given that some gene copies were barely expressed during development, they might be functionally redundant. Comparison of 24 cytochrome P450 genes associated with carotenoid biosynthesis revealed the tandem or proximal duplication resulting in expansion of *CYP* gene family. These results provided molecular information for carrot carotenoid accumulation and contributed to a new genetic resource.

## Introduction

Carrot is a biennial herb of the Apiaceae family that originated in Afghanistan and is considered an outstanding source of vitamin A owing to its high carotenoid content [1, 2]. Carotenoids in carrots can provide substrates for vitamin A and contribute to the prevention of cancers, the regulation of blood sugar levels, fighting oxidation, and slowing aging [3–5]. Carrot roots also stores carbohydrates, vitamins, dietary fiber, and other metabolites and nutrients [1]. Fleshy color is one of the most important characteristics of carrots. The fleshy roots of carrots vary in color due to the variety of carotenoids and anthocyanins they contain [6, 7]. The color change of fleshy roots is one of the hallmarks of carrot evolution, and the genetic diversity of carrots has been continuously enriched through human domestication and natural selection. Studies have shown that insertions in the MYB transcription factor caused the purple-to-yellow transformation of anthocyanin-type carrot roots [7, 8]. However, few studies have elucidated the pigmentation mechanism of carotene-type carrots with yellow, orange, red, or white roots.

Molecular regulation studies are crucial for elucidating the mechanisms of carotenoid accumulation [9, 10]. As the main source of carotenoids in nature, carrots are used as model plants to explore the molecular mechanisms of synthesis of these molecules [11–13]. Previous studies have shown that *Y*, *Y_2_* and *L* loci in carrots determine the variation in carotenoid-related phenotypes in roots [14, 15]. However, besides the candidate gene identified at the *Y* loci [14], other key genes are not well understood. Several rate-limiting enzymes are involved in carrot carotenoid synthesis; although the functions of their coding genes have been preliminarily verified [11–13, 16], a large gap remains to be filled. Current genetic information on carrots is incomplete, limiting the development of molecular mechanism research.

Telomere-to-telomere (T2T) genome refers to high-quality gapless genomes obtained by high-depth sequencing and assembly using multiple sequencing platforms [17, 18]. The assembly of T2T genomes enables the acquisition of the complete genome of species, which is conducive to an in-depth study of the molecular mechanism of character variation [19]. In recent years, the T2T genomes of many horticultural plants have been released [20–22], but that of carrot is not yet available. Currently, the most commonly used carrot genome is *Daucus carota* v2.0, which is based on the doubled haploid “Nantes” line and sequenced using an Illumina platform [14]. Although the high-throughput next-generation sequencing platform has greatly optimized the genome quality, genomes assembled using next-generation sequencing data have many unknown regions and poor integrity. There are nine main types of orange carrots with different genetic backgrounds around the world, i.e. “Amsterdam”, “Berlicumer”, “Nantes”, “Danvers”, “de Colmar”, “Chantenay”, “Paris Market”, “Imperator”, and “Kuroda” [23]. The genetic information of many carrot genotypes is unknown; a gapless high-quality carrot genome will help advance research on species evolution, genetics, and breeding.

**Figure 1:**
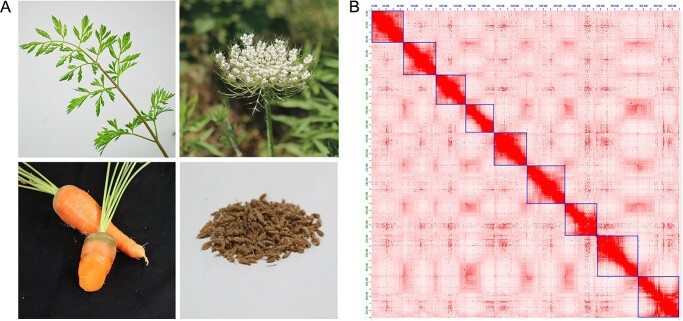
Overview of the *D. carota* vT2T genome. A. Different “Kurodagosun” carrot organs. B. Hi-C heat map of carrot chromosome interactions. Blue boxes represent individual chromosomes.

Here, we assembled a *D. carota* T2T gapless genome of the high generation inbred line of carrot “Kurodagosun” by combining the data of Oxford Nanopore Technology (ONT) Ultra-long and PacBio HiFi (high fidelity) with the aid of high-throughput chromosome conformation capture (Hi-C). The “Kuroda” type carrot is an improved variety, cultivated by Japanese breeders, which resulted from crossing European and Asian varieties [23, 24]. It is widely cultivated in China, Japan, and Korea, and is the main type used for large-scale cultivation in China (Fig. 1A). Gene annotation and comparative genomic analyses were performed based on the obtained carrot genome. Furthermore, carotenoid metabolic pathway genes in the carrot T2T genome were excavated by homology alignment, and the carotenoid-related cytochrome P450 gene family members were further analyzed. The results of this study compensated for the limitations of many gaps and unknown regions in the carrot genome. A complete carrot genome with high continuity, integrity, and accuracy was obtained, providing a genetic basis for further molecular research on carrot.

## Results

### *D. carota* T2T genome

#### Genome assembly

For the preliminary identification of carrot samples, next-generation sequencing and *K*-mer analysis were performed to estimate genome size and heterozygosity. The depth frequency distribution of the samples was analyzed based on *K*-mer = 19. As shown in Fig. S1 and Table S1, the genome size of “Kurodagosun” carrot was about 427.33 Mb, and it showed a heterozygosity rate of 0.6%.

After ONT Ultra-long, PacBio HiFi, next-generation, and Hi-C sequencing, 35.12 Gb, 36.14 Gb, 650.94 Mb, and 66.27 Gb pass reads were obtained. The detailed statistics of the sequencing data are shown in Tables S2–S5. Three gaps were found in the genome that was initially assembled using the original ONT Ultra-long and HiFi data (Tables 1 and S6). By sequence alignment, 111, 95, and 105 PacBio HiFi reads were matched to these gaps on chromosomes 1, 4, and 9, respectively. Among the ONT Ultra-long sequences, 86, 76, and 82 reads could be aligned to the gaps given that most of them aligned with both ends of the gap they covered; the mapping locations for these reads are shown in Fig. S2. Finally, a T2T level genome with zero gaps in each chromosome was generated. A 430.40 Mb sequence covered all nine chromosomes, which is longer than that of *D. carota* v2.0 (Table 2). The GC content of the *D. carota* vT2T genome was slightly lower than that reported in the previous version, and a higher contig N50 of up to 45.71 Mb was also obtained. Figure 1B shows a Hi-C heat map of the finally assembled T2T genome chromosomes. The interaction intensity within each chromosome was high, and no abnormal interaction signals were detected, indicating that the Hi-C assisted assembly results of the carrot T2T genome were correct.

**Table 1:** Statistics of gap filling of *D. carota* vT2T assembly

Gap ID	Position	Number of coverage reads			Sequence source
		Total reads	Complete coverage reads	Partial coverage reads
Gap 1	Chr1:	111	82	29	PacBio HiFi
	54 911 210 ~ 54 912 147	83	79	4	ONT Ultra-long
Gap 2	Chr4:	95	66	29	PacBio HiFi
	1 703 746 ~ 1 704 673	76	73	3	ONT Ultra-long
Gap 3	Chr9:	105	76	29	PacBio HiFi
	35 466 679 ~ 35 467 613	82	78	4	ONT Ultra-long

**Table 2:** *D. carota* genome assembly statistics

Parameter	*D. carota* vT2T	*D. carota* v2.0
Genome size (Mb)	430.40	421.50
Contig N50 (Mb)	45.71	31.23
Number of scaffolds	9	4826
Number of telomeres	15	0
Number of centromeres	9	0
GC content (%)	34.80	36.04
BUSCOs (%)	98.90	92.00

#### Genome evaluation

The reads obtained by next-generation sequencing were aligned with the assembled genomes to assess genomic consistency (Table S7). The mapping rate was 99.74% with a coverage rate of 99.97%. A BUSCO assessment of the genome was performed to verify the integrity of the genome assembly (Table S8). The results showed that 98.9% of the conserved genes were matched, of which 1529 were single-copy homologous genes and 68 were multi-copy homologous genes, indicating good overall genomic assembly integrity. The results of the *K*-mer statistical analysis showed that the QV value of the genome was 53.27, and the QV value of each chromosome was between 48.84 and 59.53 (Table S9), indicating high accuracy of the genome assembly.

#### Genome annotation

Based on different prediction methods, repeat sequences in the carrot T2T genome were annotated and the results for TE Proteins and *de novo + RepBase* were obtained. After combining the prediction results and eliminating redundancy, 238.01 Mb of duplicated sequences were obtained, accounting for 55.30% of the entire carrot genome. The main type of repeats was long terminal repeats (LTRs), which accounted for 30.3% of the whole genome, including LTR-Gypsy (7.63%) and LTR-Copia (18.22%) (Table S10).

**Figure 2:**
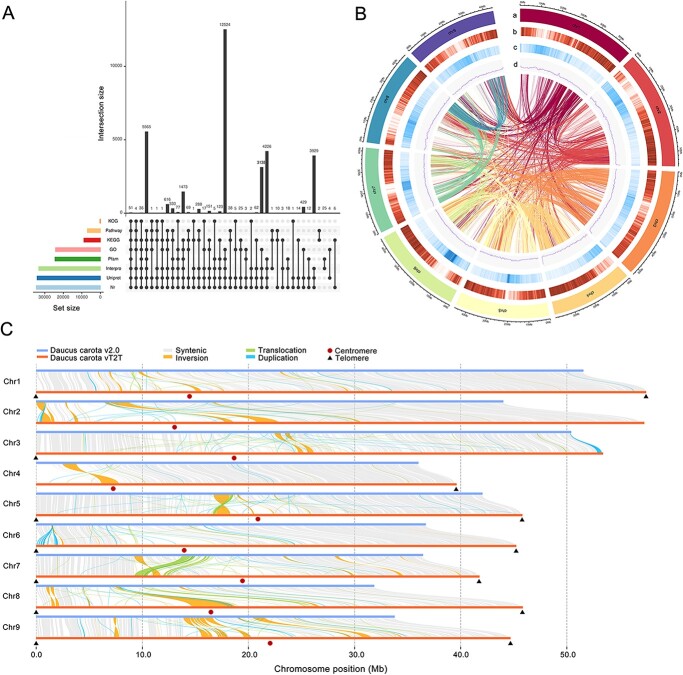
High-quality T2T carrot genome assembly. A. Gene function annotation of *D. carota* vT2T genome. B. Distribution of *D. carota* vT2T genomic features: (a) assembled chromosomes, (b) gene density, (c) repeats density, and (d) GC content (window size = 50 kb). Lines in the center of the circle indicate syntenic blocks. C. Structural variations between *D. carota* v2.0 and vT2T genomes.

*De novo* prediction, homology prediction (compared with *Apium graveolens*, *Coriandrum sativum*, *Angelica sinensis*, and *Oenanthe sinensis* genomes), and transcriptome prediction (next-generation and third-generation transcriptome) were combined to determine the genetic structure of the carrot genome, the results were integrated and de-redundant to the final data set (Table S11). In total, 36 268 genes, 195 037 exons, and 158 769 introns were identified in the carrot genome. According to the annotation information, the average mRNA, CDS, exon, and intron lengths were 4630.72 bp, 1202.11 bp, 301.98 bp, and 684.09 bp, respectively, with an average exon number of each gene was 5.38. The length distribution of genes, CDS, exons, and introns in the carrot genome was compared with those of celery, coriander, angelica, and water fennel (Fig. S3). The results showed that the frequency distribution of gene and intron lengths in the carrot genome was basically similar to that of other species, except for some differences with the gene length distribution in the angelica genome. Multiple protein databases were used to compare the protein sequences encoded by the predicted genes (Table S12), and 34 961 of them (96.40%) matched the database and obtained at least one annotation. A total of 8874 were annotated into KEGG, 34 562 into Nr, 34 132 into UniProt, 24 189 into GO, 202 into KOG, 24 468 into Pfam, and 33 238 into InterPro public databases, respectively (Fig. 2A).

A total of 500 tRNAs with a total length of 38 263 bp were annotated in the carrot genome based on their structural characteristics. According to Rfam and other rRNA databases, the carrot genome contained 123 miRNAs with an average length of 127 bp, accounting for approximately 0.0036% of the entire genome. In addition, 3196 and 1544 different types of rRNA and snRNA were annotated, accounting for 0.0905% and 0.0426% of the carrot genome, respectively (Table S13).

### Identification of telomeres and centromeres

Based gene density, repeat density, and GC content, a circos map of *D. carota* vT2T genome was drawn (Fig. 2B). Compared with *D. carota* v2.0 genome, the collinear analysis diagram shows a structural rearrangement of chromosomes in the two versions of the carrot genomes (Fig. 2C). These results indicated that the carrot vT2T genome filled numerous gaps in the previous version of the genome. In addition, a series of structures suggesting inversion, translocation, and duplication structures were discovered. Telomere sequence identification was performed based on repetitive sequences (TTAGG) in the telomeric region. Only 14 telomeric regions were predicted among the 9 chromosomes, mainly due to the fact that telomeric repeats were not detected on chromosome 2 and were only detected on the one end of chromosomes 3 and 4.

Centromeric regions were predicted based on the sequence characteristics of continuous high short tandem repeat density and low gene density. The locations of the centromeric regions of the nine chromosomes are listed in Table 3. These regions, most of which were not assembled in the previous version of the carrot genome, were estimated to be between 1700 and 7000 kb in length.

**Table 3:** Centromeres in *Daucus carota* vT2T genome

Chromosome	Start	End	Length (bp)
chr1	12 800 000	14 900 000	2 100 000
chr2	12 200 000	16 000 000	3 800 000
chr3	17 700 000	20 200 000	2 500 000
chr4	4 600 000	10 500 000	5 900 000
chr5	20 600 000	22 300 000	1 700 000
chr6	9 900 000	16 900 000	7 000 000
chr7	17 500 000	22 600 000	5 100 000
chr8	13 900 000	17 900 000	4 000 000
chr9	20 300 000	23 200 000	2 900 000

### Comparative genomic analysis

**Figure 3:**
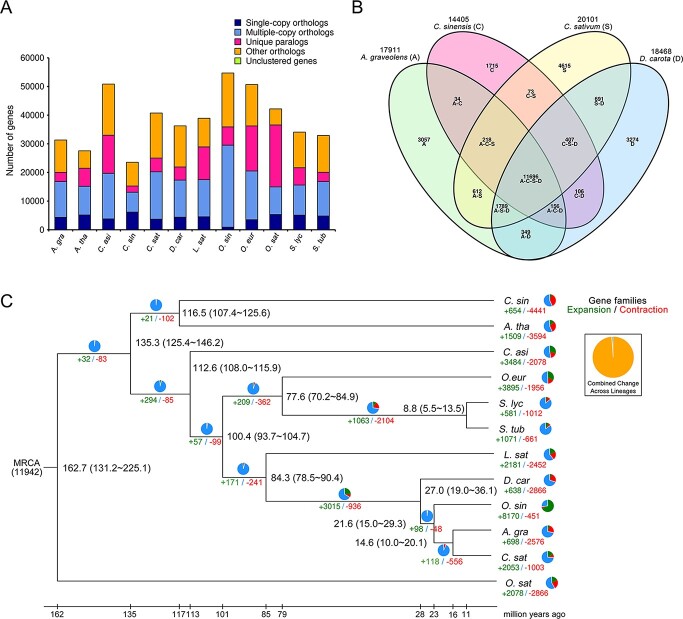
Comparative genomic analysis of the carrot vT2T genome. A. Number of homologous genes shared by different species. B. Venn diagram of gene family clustering. The numbers represent the number of gene families. The letters in parentheses are the initials used to identify each species. Linked letters indicate the gene families shared by the indicated species, whereas single letters represent gene families specific to one species. C. Estimation of divergence time and gene family expansion/contraction. Black numbers next to each branch node represent the estimated divergence time; green and red numbers represent the expansion and contraction of gene families, respectively.

Together with carrot, 11 species genomes (*A. gra*, *Apium graveolens*; *A. tha*, *Arabidopsis thaliana*; *C. asi*, *Camellia sinensis*; *C. sin*, *Citrus sinensis*; *C. sat*, *Coriandrum sativum*; *L. sat*, *Lactuca sativa*; *O. sin*, *Oenanthe sinensis*; *O. eur*, *Olea europaea*; *O. sat*, *Oryza sativa*; *S. lyc*, *Solanum lycopersicum*; *S. tub*, *Solanum tuberosum*) were selected for identifying homologous genes, gene family clustering analysis, and the enrichment of single copy genes and multiple copy genes (Fig. 3A). A total of 72 819 orthologous gene families were detected in all species, including 463 764 genes; 8745 gene families were shared by all species, including 217 207 genes; and 165 were single-copy gene families. The carrot genome consisted of 2711 unique gene families, including 4614 unique paralogs. GO and KEGG enrichment (Figs S4 and S5) of the unique carrot gene families indicated that these genes were mainly related to cysteine-type peptidase activity, protein heterodimerization activity, DNA repair, and lipid binding *etc*. (GO enrichment); and homologous recombination, DNA replication, nucleotide excision repair, and mismatch repair *etc*. (KEGG enrichment). Two closely related species, celery (*A. graveolens*) and coriander (*C. sativum*), and an outgroup species citrus (*C. sinensis*), were selected for gene family comparisons (Fig. 3B). The results suggested that 11 696 gene families were shared among these species. Carrots shared 13 990 and 14 583 gene families with celery and coriander, respectively. There were slightly fewer similarities with citrus, with 12 365 gene families in common. In particular, carrot held 3274 unique gene families.

### Phylogeny of carrot

A phylogenetic tree was constructed to estimate the divergence time of the 12 species (Fig. 3C). Apiaceae plants were diverged from lettuce in Compositae approximately 84.3 million years ago (Mya), and from the cluster of celery and other Apiaceae species at around 27.0 Mya. The common ancestor of the selected species was estimated to have 11 942 gene families (Fig. 3C). In total, 2866 gene family contractions and 638 gene family expansions were detected in carrot, which were most closely related to those in celery (2576 gene family contractions and 698 gene family expansions). In contrast, water fennel and coriander, which are also members of the Apiaceae family, had more expansion gene families. The most conspicuous contraction gene family was mainly related to plant hormone signal transduction, while the expansion gene family was involved in ubiquitin mediated proteolysis and endocytosis (Figs S6–S9).

### Whole-genome duplications of carrot

**Figure 4:**
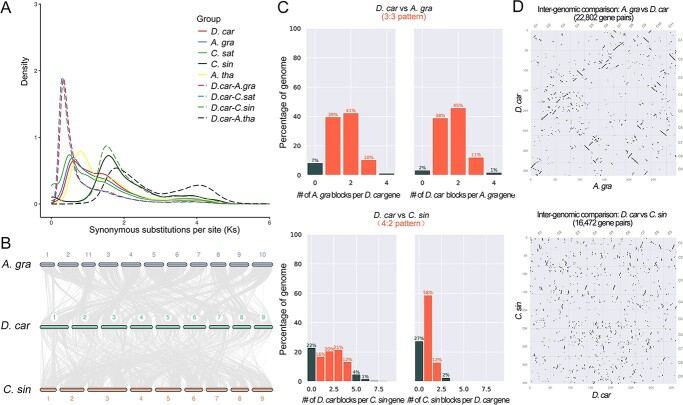
Genome evolution of carrot vT2T genome. A. WGD analysis diagram. B. Collinearity diagram including *D. car*, *A. gra*, and *C. sin*. C. Syntenic depth ratio analyses of *D. car* vs. *A. gra* and *D. car* vs. *C. sin*. D. Scatter plots of *D. car* vs. *A. gra* (upper panel) and *D. car* vs. *C. sin* (lower panel).

Based on a synonymous mutation rate analysis of homologous genes, two whole-genome duplication (WGD) events occurred in carrot. A comparison between celery and coriander genomes confirmed that these species experienced a hexaploidization event (WGT) and a WGD event shared by Apiaceae plants (Fig. 4A). Among these three Apiaceae species, the different *Ks* values indicated that carrot evolved first, followed by celery and coriander. Furthermore, collinearity analysis of the carrot and celery genomes was performed with an overall syntenic depth ratio of 3:3 (Figs 4B–D), indicating that both species experienced the same WGD events. In contrast, collinearity analysis with the citrus genome indicated that the syntenic depth ratio was approximately 4:2.

### The carotenoid metabolic pathway in carrot

#### Structural genes in the carotenoid metabolic pathway

**Figure 5:**
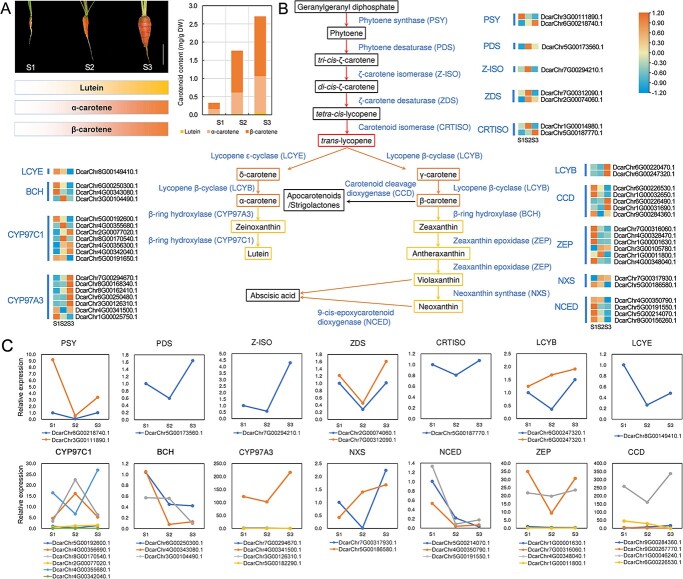
Carotenoid metabolic pathway in carrot. A. Carotenoid accumulation in fleshy roots of carrot at different developmental stages. S1, S2, and S3 represent 40, 60, and 90 DAS, respectively. B. Carotenoid pathway and related gene expressions in carrot. The heatmaps indicate the gene expression levels at different developmental stages (S1, S2, and S3). C. Relative expression levels of carotenoid pathway genes in carrot.

The changes in the carotenoid content of the roots of carrot “Kurodagosun” during development were measured. Increasing concentrations of lutein, α-carotene, and β-carotene were detected throughout root developmental stages. At 90 days after sowing (DAS), the total carotenoid content was more than eight times that at S1, reaching concentrations of 2.71 mg/g dry weight (Fig. 5A).

We then reconstructed the carotenoid metabolic pathway affecting fleshy root coloration in carrots based on the carrot T2T genomic data (Fig. 5B, Table S14). In total, 65 genes were identified as structural genes in the carotenoid metabolic pathway by homology comparison and screening. Among them, 56 genes could be aligned with genes in *D. carota* v2.0 genome and the other 9 were newly annotated in the *D. carota* T2T genome. The genes obtained by screening were mainly concentrated on chromosomes 1, 4, 5, and 6, with 11, 11, 12, and 9 genes, respectively. Both *PDS* and *LCYE* genes are single-copy genes, whereas all other carotenoid pathway genes had multiple copies, either on the same or different chromosomes. A total of 24 carrot genes belong to the cytochrome P450 gene family; they are similar to the *CYP97C1*/*lut1* and *CYP97A3*/*lut5* genes in *A. thaliana*. The copy numbers of the zeaxanthin epoxide gene (*ZEP*), carotenoid cleavage dioxygenase gene (*CCD*), and 9-*cis*-epoxycarotenoid dioxygenase (*NCED*) genes were also relatively high.

Based on the annotated repeat sequences in the *D. carota* T2T genome, we screened for transposons that overlapped with the carotenoid synthesis pathway genes. Different numbers of transposons were identified in the vicinity of *PSY*, *PDS*, *CRTISO*, *LCYE*, *BCH*, six *CYP97C1*, *CYP97A3*, four *CCD*, and two *NXS* genes. These transposons included LTR-Copia, LTR-Gypsy, LTR-MULE, LTR-hAT, and many other types, as well as some unknown categories (Table S15).

#### Transcription of carotenoid structural genes

We compared the transcript levels of the carotenoid structural genes, and a lot of which showed no detectable expression (Fig. 5B). The relative expression levels of these genes were further verified by quantitative PCR to obtain accurate expression trends. The comparison of two *PSY* genes showed that DcarChr6G00218740.1 was highly expressed. Most of the expressions of desaturase genes (*PDS* and *ZDSs*) and isomerase genes (*Z-ISO* and *CRTISO*s) were at its lowest at S2. The transcript levels of the lycopene cyclase gene *LCYE* decreased first then increased with growth and development, while those of two *LCYB* genes differed. The expression level of DcarChr6G00247320.1 expressed higher and increased gradually. The carotene hydroxylase genes were divided into three clusters: *CYP97A3/lut5*, *CYP97C1/lut1*, and *BCH*. There were three *BCH* genes, two of which had similar downregulated expression trends. The expressions of most cytochrome P450 family genes were quite low. Gene DcarChr4G00341500.1 was highly expressed. Among the 9 *CCD* genes that were identified, which include several proximal duplications, only four (DcarChr6G00226530.1, DcarChr1G00032650.1, DcarChr6G00226490.1, and DcarChr1G00031690.1) were expressed and DcarChr1G00032650.1 was the predominant gene expressed. Two *ZEP* genes were highly expressed and the expression level of DcarChr4G00348040.1 was stable in the three development stages. The expression of two *NXS* genes showed opposite trends. *NCEDs*, which control the transformation of neoxanthin into abscisic acid (ABA), were highly expressed during the early stages of carrot growth.

### Analysis of the cytochrome P450 genes associated with carotenoid biosynthesis

**Figure 6:**
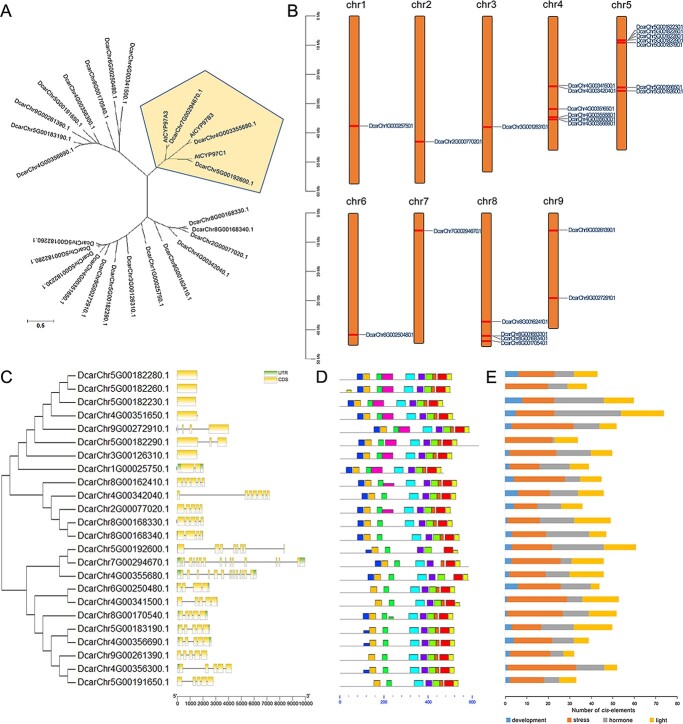
Cytochrome P450 genes related to carotenoid metabolism in carrot. A. Phylogenetic tree of carrot carotenoid-related cytochrome P450 genes and *Arabidopsis CYP97* genes. The lower left line represents genetic distance. B. Gene location of carrot carotenoid-related cytochrome P450 genes on carrot chromosomes. C. Gene structure of carrot carotenoid-related cytochrome P450 genes. D. Predicted motifs in carrot carotenoid-related cytochrome P450 genes. E. Statistics of the *cis*-elements identified in the promoter regions of carrot carotenoid-related cytochrome P450 genes.

A total of 24 cytochrome P450 genes were identified by homology comparison with *CYP97* gene family members of *A. thaliana* (*AtCYP97A3*, *AtCYP97B3*, and *AtCYP97C1*), and further analyses of these genes were conducted (Fig. 6). A phylogenetic tree showed that these genes were distributed into three lineages, and that three of the genes (DcarChr7G00294670.1, DcarChr4G003355680.1, and DcarChr5G00192600.1) were closely related to the *AtCYP97* genes (Fig. 6A). Figure 6B shows the locations of the cytochrome P450 genes on carrot chromosomes. Tandem and proximal duplications were observed on chromosomes 4, 5, and 8. According to the genomic annotation information, five of these *CYP* genes had only one CDS, while the remaining had 2 ~ 15 (Fig. 6C). The motif prediction results (Figs 6D and S10) showed that the 24 carotenoid synthesis-related *CYP* genes shared seven motifs (motifs 1, 3, 4, 5, 6, 7, and 10), and that 10 of these genes contained up to ten motif sequences. In order to further elucidate the potential functions of these genes, the *cis*-acting elements in their promoter regions were analyzed (Fig. 6E). These genes were predicted to contain different numbers of developmental, stress, hormone, and light responsive elements. Among them, the numbers of stress response elements and hormone response elements was relatively high.

## Discussion

Carrot is an important root vegetable whose fleshy roots contain various nutrients, including carotenoids, carbohydrates, and dietary fibers, *etc.* [1]. Because of its rich carotenoid content and types, it is considered to be one of the main sources of vitamin A for the human body [25]. In a previous study, the carrot genome was assembled at the chromosome level, but the tested material was the western carrot “Nantes” [14]. Given the different evolutionary and transmission patterns of the western and eastern types of carrots, the lack of high-quality carrot genome sequences of different varieties remains to be resolved. The assembly of the T2T genomes of various horticultural crops has assisted in the identification of key traits [26]. The “Mca genome” of bitter melon (*Momordica charantia*) helped to explore the molecular regulation mechanism of ripening and flavor formation during fruit growth and development [21]. The near-complete genome of *Brassica rapa* revealed the rapid evolution of the centromeric region of Chinese cabbage [27]. Using a gapless genome combined with multi-omics studies, the regulatory mechanism of azalea color variation was identified [22]. In order to better use the genome to reveal the evolution law and genetic variation of different carrot types, we built a high-quality T2T carrot genome based on the high generation inbred line of carrot “Kurodagosun”. The results not only increased the understanding of the oriental carrot genome but also revealed many previously unknown genomic regions in *D. carota*.

In this study, the first complete genome with 9 chromosomes of carrot “Kurodagosun” was constructed by optimizing various strategies, and the genome size was 430.40 Mb, which was slightly larger than that of the previously reported genome belonging to “Nantes” carrot [14]. The results of the collinear analysis showed many structural variations including inversions, translocations, and duplications in the genomes of the two carrot varieties. These variations could reflect actual differences between the two carrot varieties, but may also be due to technical improvements in the determination of the genome. The telomeres and centromeric regions on the 9 carrot chromosomes were predicted. Comparatively, the number of annotated genes increased from 32 113 to 36 268 [14]. These new findings provide a theoretical basis for molecular marker-assisted breeding and genetic function research on carrots.

To better understand the evolution of *D. carota*, the evolutionary relationships between carrots, Apiaceae plants, and some peripheral species were compared. Previously, Iorizzo et al. proved that the divergence between carrots and lettuce occurred at approximately 72.0 Mya [14]. However, based on the fossil time node, our results indicated that Apiaceae plants separated from lettuce about 84.3 Mya. Elucidation of the celery genome revealed two subsequent polyploidization events in Apiaceae species [28]. Our analysis supported this conclusion and suggested that carrots evolved earlier than the other Apiaceae species analyzed here. *D. carota* diverged from other Apiaceae species 19.0 ~ 36.1 Mya. These results can serve as a reference for other Apiaceae genome studies.

Fleshy root coloration of carrots depends largely on the type and content of carotenoids [29]; however, the molecular mechanisms underlying carotenoid accumulation in carrots are not well understood. The carrot carotenoid metabolic pathway was reconstructed in this study. Many structural genes have been found to have multiple copies after genome-wide replication; however, transcriptome data indicated that some of them were not transcribed. Previous studies have demonstrated that some of these multi-copy genes play roles in different carrot tissues [30], and that functionally redundant genes are also present [13]. Some genes maintained high transcription levels among multiple copies, indicating that they may play a major role in these duplications. However, the activity of enzymes encoded by these genes cannot be ignored: when the enzyme holds high activity, there is no need to have excessive expression. Nine of the carotenoid pathway genes identified in this study were newly annotated genes in carrots, providing novel insights into the carotenoid pathway. The functional verification of these genes in the future can better elucidate the mechanism of carotenoid synthesis.

The T2T assembly strategy enables the reconstruction of a high continuity and integrity genome and facilitates the acquisition of information on complex repeat sequence regions. A total of 30.30% of LTRs were obtained from the carrot genome, including LTR-Gypsy (7.63%) and LTR-Copia (18.22%). LTR transposons are important repeat sequences that affect the genome in many aspects. When transposons are inserted into key functional genes, they can cause changes in plant traits, and when inserted into other regions, they can regulate gene expression [31]. Increasing or decreasing the number of LTR transposons also causes changes in genome size [29]. In carrots, a key transcription factor, DcMYB7, was transcriptionally inactivated after being interrupted by two transposons, resulting in the loss of anthocyanin accumulation in purple carrots [7]. The discovery of carrot LTR transposons will be helpful for further research on the molecular mechanisms of key characteristics of the plant. Transposons near the carotenoid pathway genes were screened based on the annotation of repeat sequences. Evidence has shown that LTRs may drive the expansion of duplicated genes [32]. The existence of these transposons can promote the differentiation of gene functions and lead to the generation of new traits, which may be a good entry point for research on carrot carotenoid gene function.

Members of the cytochrome P450 gene family, which are involved in carotenoid synthetic, are of particular interest. Cytochrome P450 enzymes, one of the largest enzyme protein families in higher plants, are involved in a variety of metabolic reactions and play important roles in the synthesis of lignin [33], plant hormones [34], flavonoids [35], and terpenoids [36]. A total of 24 cytochrome P450 genes were identified by homology comparisons, and 3 of them were closely related to *Arabidopsis CYP97* genes. One of these *CYP97*-like genes, DcarChr7G00294670.1, was previously identified in carrot as *CYP97A3*; it plays a role in β-ring hydroxylation. It was noted that the *DcCYP97A3* gene contained a frame-shifting insertion in orange carrots, which affects the α-carotene content [16]. Lutein is produced under the action of another gene, *CYP97C1* (DcarCh5G00192600.1) [37]. However, the functions and mechanisms of other cytochrome P450 genes require further exploration. Interestingly, cytochrome P450 genes underwent tandem and proximal duplications on chromosomes 4, 5, and 8, and research on these genes could further help to understand genetic diversity during evolution.

## Materials and methods

### Plant materials

The leaves of the “Kurodagosun” carrot inbred line were selected as the material for genome analysis. This inbred line was obtained by forced selfing over successive generations. The plants were cultivated in the plant growth room of the State Key Laboratory of Crop Genetics & Germplasm Enhancement and Utilization, Nanjing Agricultural University. Leaves from a single carrot with good growth conditions at 60 DAS were sampled for DNA sample extraction and subsequent genome determination. Carrot plants at 40 (S1), 60 (S2), and 90 (S3) DAS were used to measure carotenoid contents. More than three independent plants were selected for mixing during each period.

### Genome sequencing

#### ONT ultra-long sequencing

The Nanopore sequencing platform was used for the Ultra-long sequencing of DNA samples. The failed reads were removed from the raw data and Filtlong v0.2.4 software (https://github.com/rrwick/Filtlong) was used to filter the fragments <10 kb. The obtained pass reads were used for subsequent analyses. The joint sequence was filtered using Porechop v0.2.4 software, reads with retention lengths ≥30 kb and mean read quality scores >90% were used for assembly.

#### PacBio HiFi sequencing

HiFi sequencing was performed using the PacBio platform, and the quality of the original data was evaluated using CCS v6.0.0 software. Data sequenced in less than three cycles, low-quality subreads, and data with signal-to-noise ratios <2.5 were filtered out. Effective circular consensus sequencing (CCS) reads with high accuracy were obtained for subsequent analysis.

#### Hi-C sequencing

Hi-C technology was used to explore the spatial positional relationship of chromatin DNA in the genome, and clean data were obtained after removing joint and low-quality sequences from the original data. With help of HiCUP v0.8.0 software (http://www.bioinformatics.babraham.ac.uk/projects/hicup/), unmapped reads whose both ends were not uniquely aligned to the reference genome, invalid pairs with self-loops or marginal hanging, and repeats resulting from PCR amplification were removed.

#### Next-generation sequencing

Next-generation genome sequencing was performed for subsequent read-error correction. The original data were filtered using software fastp v0.21.0 to filter out reads of low-quality, too short, or excessive N reads. The heads and tails of the reads were pruned to automatically detect adapter sequences and remove contamination, as well as duplicate sequences caused by PCR amplification. All the sequencing was finished by Wuhan Benagen Technology Co., Ltd (China).

### Genome assembly

The initial assembly of the ONT Ultra-long sequencing data was performed using NextDenovo v2.5.0, NECAT, and Flye software [38]. The NextDenovo assembly parameters were as follows: read_cutoff = 1 k, blocksize = 1 g, nextgraph_options = −a 1. The NECAT parameter was defined at the default value. Flye assembly parameters were “-i 3-m 10000”. Hifiasm v0.16.1 software was used for genome assembly of the PacBio HiFi data [39]. Minimap2 software was used to compare data from mitochondria and chloroplasts, sequences with more than a 50% base alignment were removed, bacterial contamination was removed using BLAST RefSeq library, and contigs supported by low reads were removed [40].

ALLHiC software was used to cluster the contig sequences into different chromosome groups using a bottom-up hierarchical clustering algorithm [41]. The contigs within each chromosome group were ordered and oriented. The pairwise interaction between contigs was then converted into “.hic” files using 3D-DNA and juicer software, and Juicebox software was used for manual ordering and orientation [42, 43]. After removing the heterozygous sequences, 100 N were used to fill the gaps and obtain the final chromosome-level genome sequence. HiCExplorer v3.6 software was used to plot the interaction intensity and position relationship between contigs.

The *K*-mer anchoring method was used to target the centromeric region for the three-generation genomic error correction. ONT reads were aligned to pseudochromosomes using minimap2 software. Medaka consensus v1.5.0 was used for error correction, and the consistency sequence after three generations of error correction were obtained using the medaka stitch. DeepVariant v1.3.0 software was used for next-generation error correction [44]. Winnowamap v.11 was used to collect all reads within 50 bp at the end of the chromosome [45].

The telomeric repeat sequence were identified according to the “Telomere database” (http://telomerase.asu.edu/sequences_telomere.html) among those sequences that continuously appeared in the reads, the sequence that appeared most times was defined as “ref”, whereas the rest was considered the “query”. The telomeric sequences were reassembled to obtain consistent sequences and were then matched to each chromosome. Gap filling data and genome gap interval were compared, the gaps were filled at three levels according to the priority “genome version after error correction > HiFi data > ONT Ultra-long data”. Those sequences whose positions on the alignment crossed both ends of the gap were selected, and the best aligned sequence covering the longest length region was used to fill the gap region in the genome. HiFi reads were compared to gap-filled genomes. The corresponding fragments were filtered, the chimeric fragments were removed, and a special branch of Racon v1.6.0 (−L-u; https://github.com/isovic/racon/) was used for error correction.

### Genome annotation

The genome repeat sequence was predicted by the *de novo* method using RepeatModeler software, and non-redundant LTR sequences were obtained by LTR_FINDER and LTR_retriever software. The two sequences were then combined to obtain a *de novo* repeat sequence library. The *de novo* + RepBase result was obtained using RepeatMasker software after combining the sequence with RepBase library. The TE_protein repeat sequence was predicted by RepeatProteinMask software to obtain TE proteins. The final genome repeat set was obtained by combining all the repeated predictions and eliminating redundancy.

Gene structure was predicted based on transcriptome prediction, homology prediction and *de novo* prediction. After filtering, the original reads were mapped to the genome and the transcripts were reconstructed to predict the coding frame. Homology prediction was based on the protein sequence files of celery, coriander, water fennel, and angelica. *de novo* prediction was performed using Augustus software based on genomes masked by repeat sequences [46]. MAKER software was used to integrate the gene sets obtained by the three prediction methods, and the accuracy of the predicted open reading frame (ORF), starting and ending locations of the coding regions and gene lengths were verified [47]. The integrity of genome annotations was assessed using BUSCO software [48]. Based on sequence and motif similarities, the proteins were compared with UniProt [49], Nr [50], GO [51], KOG [52], Pfam [53], InterPro [54], and KEGG [55] protein databases to identify possible biological functions and obtain metabolic pathway information of the sequences. Blastp software was used for self-alignment of protein sequences [56], and then MCScanX was used to identify collinear blocks [57]. Circlize in R language was used to draw a circos diagram [58].

The tRNAs in the genome were identified according to their structural characteristics, while rRNAs were predicted by the rRNA database, and snRNA and miRNA sequences were annotated using INFERNAL software based on the Rfam database [59].

### Centromere prediction

According to the characteristics of high density of short tandem repeats and low gene density in the centromeric region, BEDTools software was used to calculate the short tandem repeats and gene coverage in the genome sequence, and the position of the centromere was predicted using a collinear circos diagram of the genome.

### Genomic evolutionary analysis

#### Gene family cluster analysis

Together with carrot, 11 plants, including rice, celery, coriander, water fennel, tomato, potato, tea, *Arabidopsis*, lettuce, olive, and orange were selected for genome evolution analysis. OrthoFinder software was used for gene family clustering based on all amino acid sequences of the species, and clusterProfiler was used for GO and KEGG analyses.

#### Phylogenetic tree construction

Multiple sequence alignments were performed based on the protein sequences of the single-copy gene families, and the number of single-copy genes was set to 165. A species phylogenetic tree was constructed using RAxML software. Based on the topological structure of the phylogenetic tree and fossil time nodes, the mcmctree program in the PAML package was used to estimate the time of species differentiation time.

#### Gene family contraction and expansion

According to the results of the species phylogenetic tree and the gene family clustering, the birth-mortality model was used to estimate the number of ancestral gene family members in each branch, and the contraction and expansion of the species gene families relative to those of the ancestors were predicted. Significant expansion or contraction was defined at *p* < 0.05, and functional enrichment analysis was performed on gene families with significant expansion or contraction.

#### Genome-wide replication analysis

Based on the results of protein sequence alignments between different species, the collinear blocks of the genome were analyzed using MCScanX. The frequency of synonymous mutation (*Ks*), frequency of non-synonymous mutation (*Ka*) and their ratio (*Ka*/*Ks*) of collinear gene pairs were calculated using the yn00 module of PAML software, and the density map was plotted using ggplot2.

#### Collinearity analysis

The last (version: 1170) software was used to compare the gene sequences of the two species to identify similar gene pairs. JCVI software was used to determine the positions of similar gene pairs in chromosomes, and all genes in collinear blocks were obtained.

### Identification of genes involved in carotenoid biosynthesis

To identify the genes involved in the carotenoid biosynthesis pathway in the carrot genome, the protein sequences of carotenoid-related genes from *A. thaliana* were selected and referenced. TBtools software was used to blast homologous genes in the carrot T2T genome sequence, and candidate genes with e-values <10e^−5^ were screened [60]. After removing duplicated genes, the accuracy was further verified by comparing the protein and conserved domains according to the Pfam database [53]. The transcription profiles of carrot carotenoid genes at different growth stages were analyzed using the database BIO2DB (http://celerydb.bio2db.com/download.html). A heatmap was drawn using the data with expression normalized to log2FPKM. Real-time fluorescence quantitative PCR was used to verify the gene expression levels, *DcActin* was selected as the reference gene [61]. Gene-specific primers are shown in Table S16. Transposons overlapping with carotenoid pathway genes were identified based on repeat sequence annotation results.

### Extraction and determination of carotenoids

Carotenoid content in the carrot fleshy roots was determined according to a previously described method [8]. Three carrot seedlings were selected from each growth stage and the fleshy roots were mixed for further determination. Three replicates were used for each measurement.

### Analysis of carrot carotenoid-related cytochrome P450 gene family

A phylogenetic tree was constructed by combining members of the carotenoid-related cytochrome P450 gene family with *AtCYP97A3*, *AtCYP97B2*, and *AtCYP97C1* from *Arabidopsis* using the adjacency method using MEGA software [62]. According to the genome annotation information, the position of each cytochrome P450 gene was labeled on the carrot chromosomes, and the gene structure was visualized using TBtools software [60]. MEME online software was used to predict the motifs of these *CYP* genes, and the number was set to 10 [63]. The promoter region of each carrot *CYP* gene with a length of 2000 bp was obtained from the carrot T2T genome sequence, and *cis*-acting regulatory elements were predicted according to the PlantCARE database [64]. The predicted *cis*-elements were divided into four functional categories: development, stress, hormone, and light response elements. A statistical histogram of the number of *cis*-elements was generated.

## Supplementary Material

Web_Material_uhad103

## Data Availability

The genome raw sequencing (including ONT Ultra-long, PacBio HiFi, Hi-C, and next-generation), assembly, and annotation data are accessible in the NCBI (https://www.ncbi.nlm.nih.gov/) database with the BioProject accession number PRJNA967095.
