# Supplementary material for: Telomere-to-telomere carrot (Daucus carota) genome assembly reveals carotenoid characteristics
Source: Hortic Res. 2023 May 10;10(7):uhad103. doi: 10.1093/hr/uhad103 (PMC10541555; doi:10.1093/hr/uhad103)
Supplement: Web_Material_uhad103 [file web_material_uhad103.zip › Supplementary figures R1.pdf]

Supplementary Figures

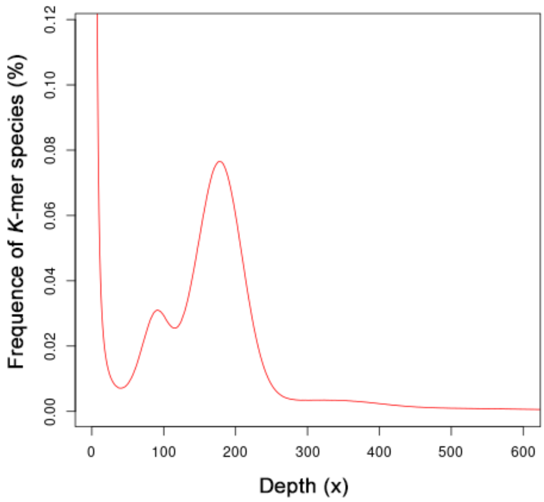

Figure S1. *K*-mer depth-frequence distribution.

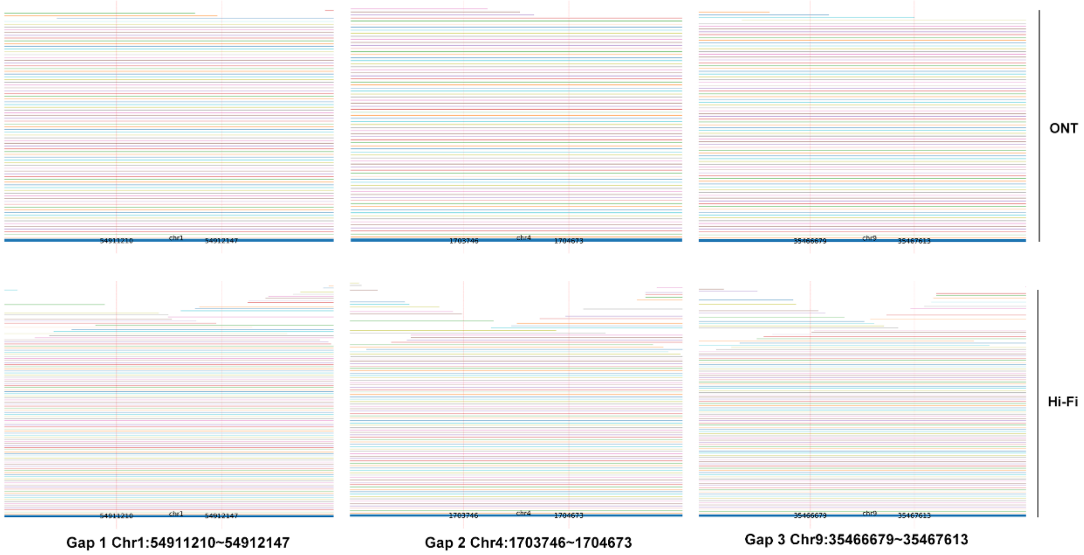

Figure S2. Mapping locations of reads for gap filling of *D. carota* vT2T.

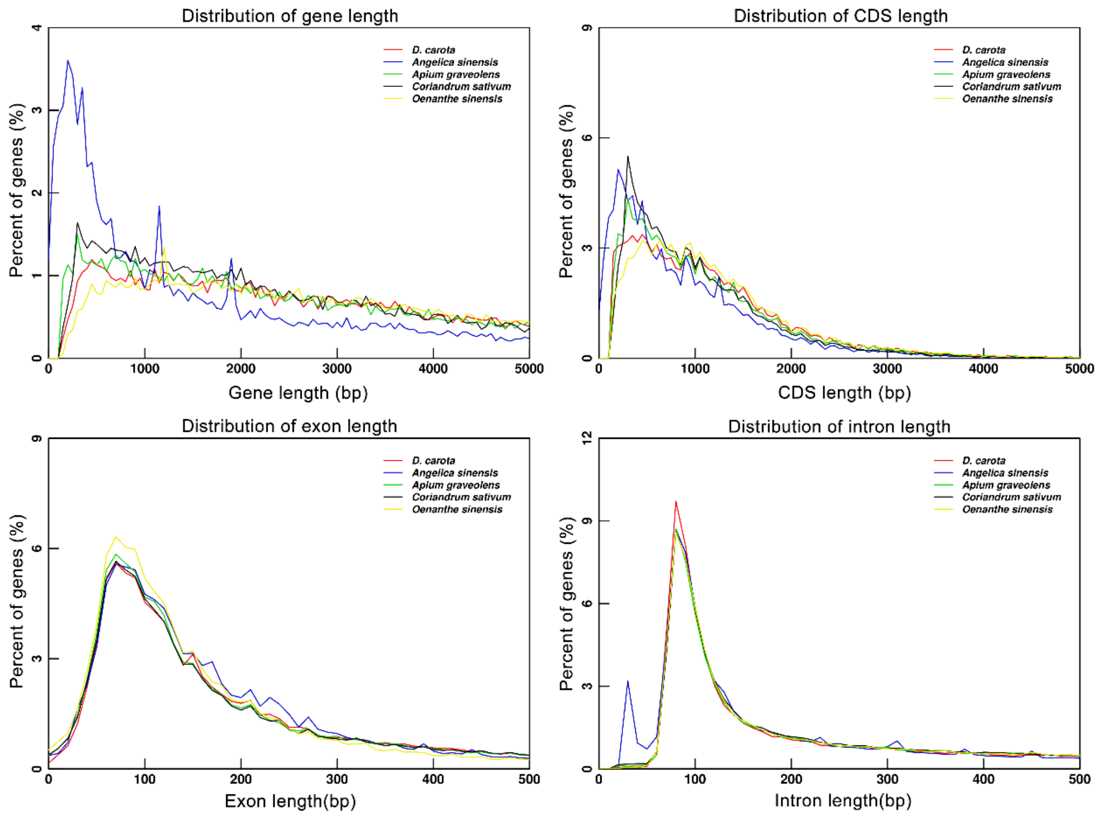

Figure S3. Comparison of gene lengths between *D. carota* and close species.

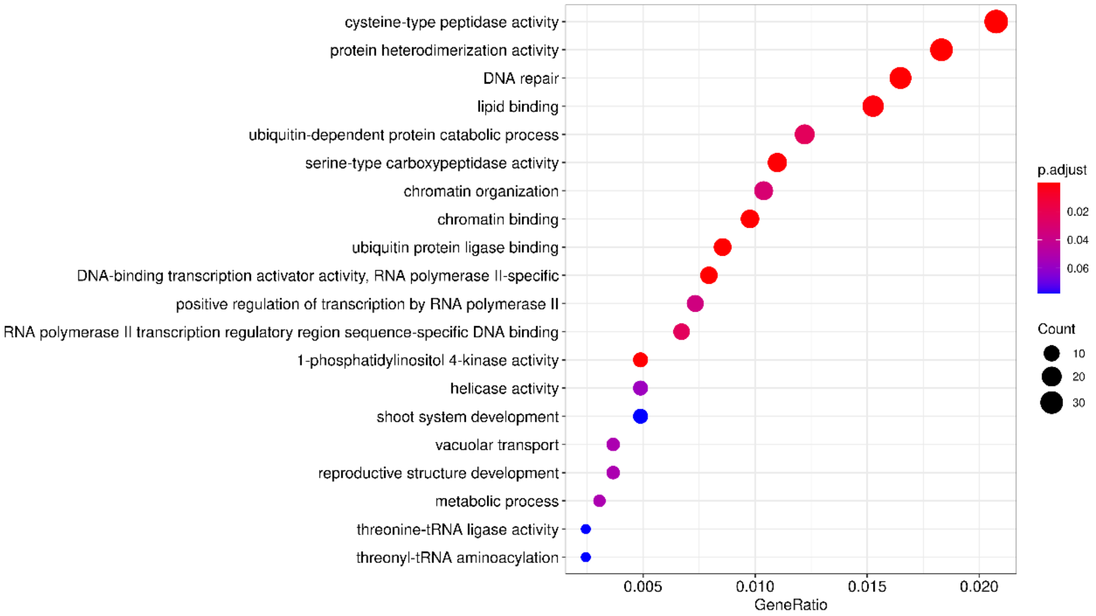

Figure S4. GO enrichment results of carrot unique gene family.

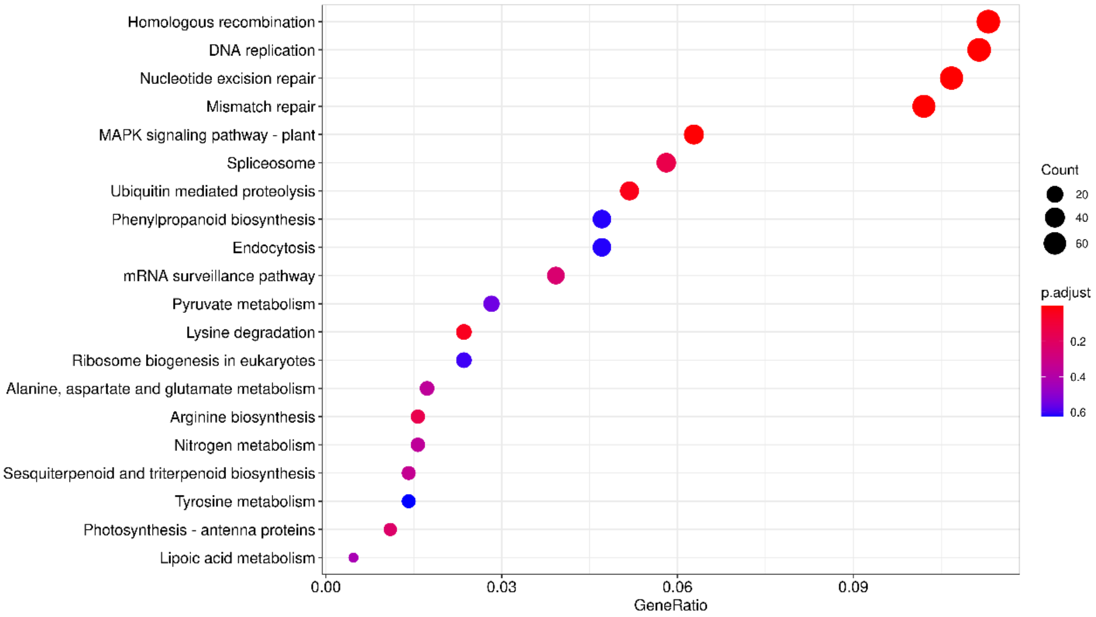

Figure S5. KEGG enrichment results of carrot unique gene family.

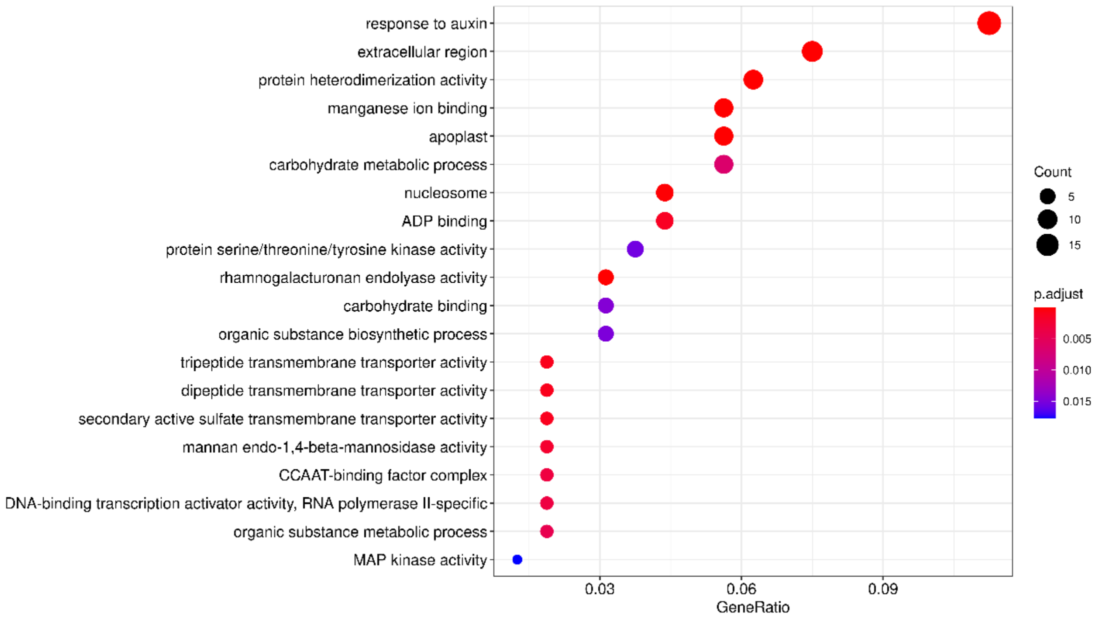

Figure S6. GO enrichment results of carrot contraction gene family.

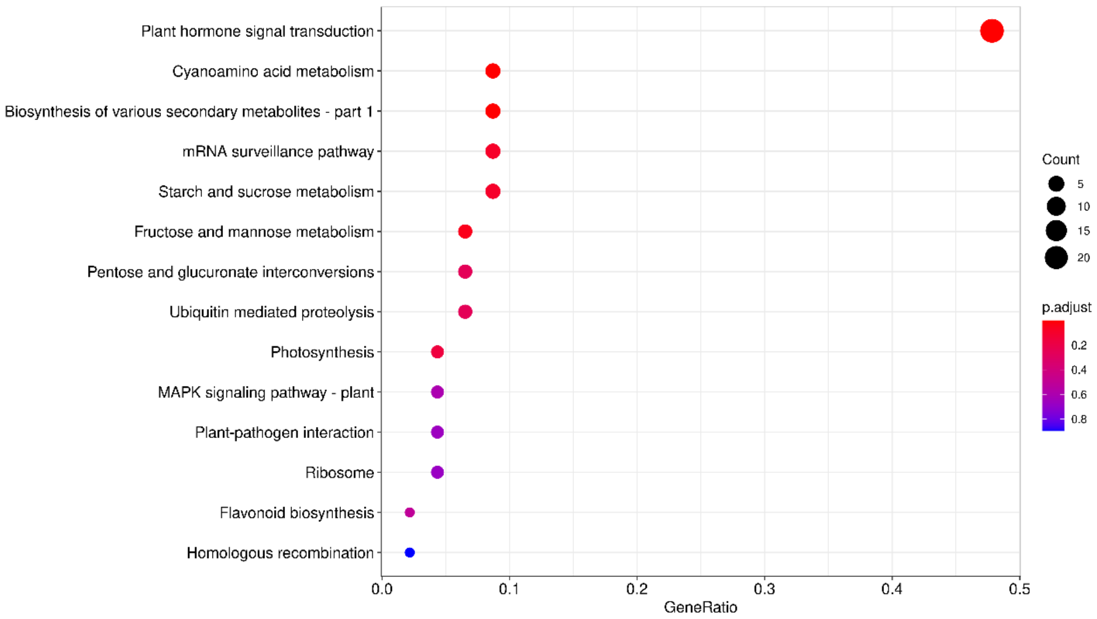

Figure S7. KEGG enrichment results of carrot contraction gene family.

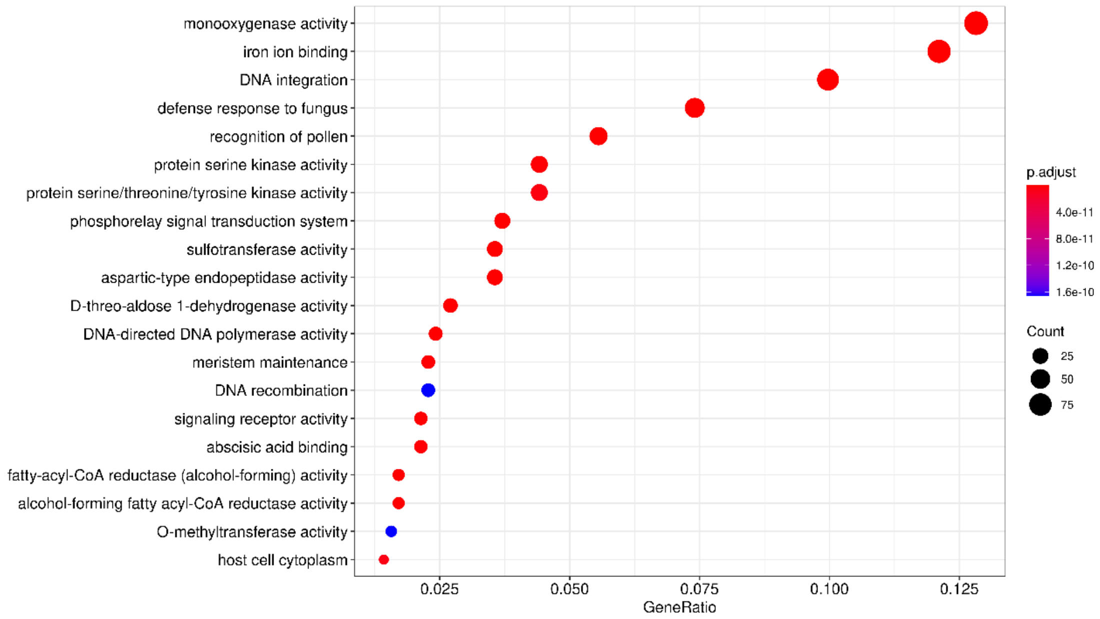

Figure S8. GO enrichment results of carrot expansion gene family.

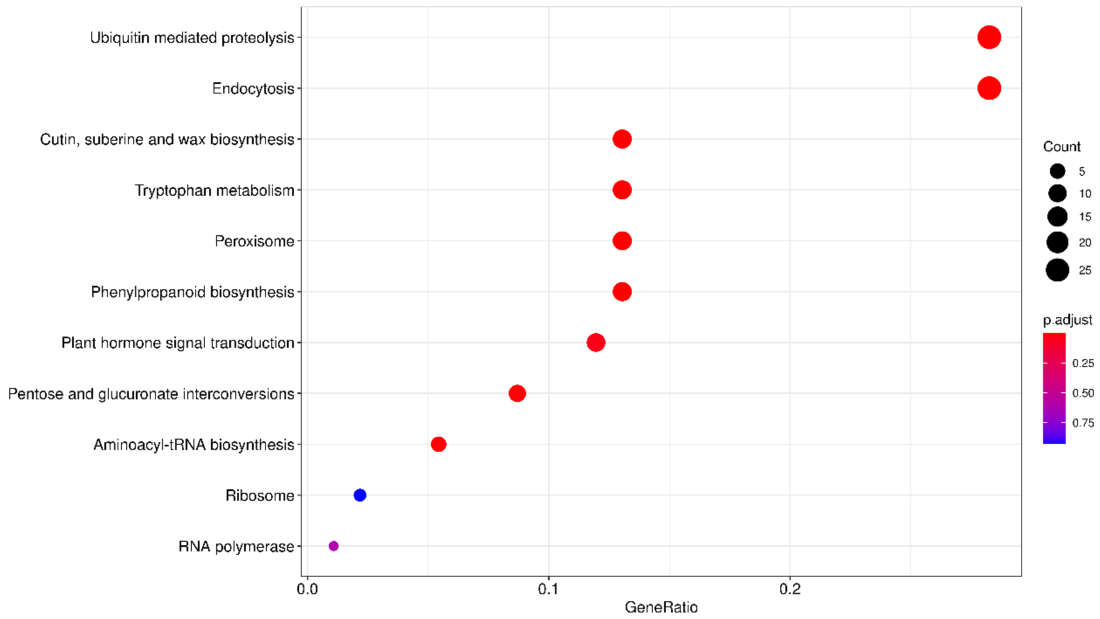

Figure S9. KEGG enrichment results of carrot expansion gene family.

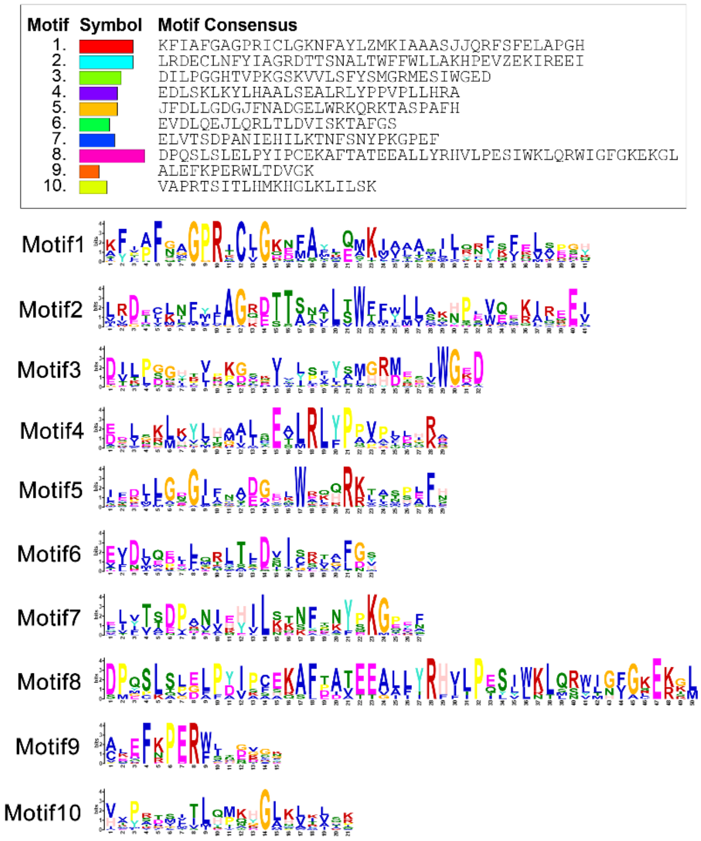

Figure S10. Motif sequences of carrot carotenoid-related cytochrome P450 gene family members.
