# Supplementary material for: Telomere-to-telomere carrot (Daucus carota) genome assembly reveals carotenoid characteristics
Source: Hortic Res. 2023 May 10;10(7):uhad103. doi: 10.1093/hr/uhad103 (PMC10541555; doi:10.1093/hr/uhad103)
Supplement: Web_Material_uhad103 [file web_material_uhad103.zip › Supplementary tables R1.pdf]

Supplementary Tables

Table S1. Results of *K*-mer analysis data.

Parameter	Value
K-mer	19
K-mer number	75,994,273,484
K-mer depth	177.83
Estimated genome size (Mb)	427.33
Data size (clean data) (Gb)	95.85
Depth	224.3
Heterozygous ratio (%)	0.6
Duplication ratio (%)	45.17

Table S2. Statistics of ONT Ultra-long sequencing data.

Rank	Flag	TotalBase	TotalReads	MaxLenth	AvgLenth	N50	L50	N90	L90	meanQ
>0	all	36,469,845,041	365,634	1,888,759	99,744.13	100,001	141,345	72,536	313,009	11.62
>0	pass	35,119,417,363	352,384	670,868	99,662.35	100,001	136,222	72,412	301,639	11.85
>0	fail	1,350,427,678	13,250	1,888,759	101,919.07	100,013	5,123	75,157	11,389	5.48
>10000	all	36,469,845,041	365,634	1,888,759	99,744.13	100,001	141,345	72,536	313,009	11.62
>10000	pass	35,119,417,363	352,384	670,868	99,662.35	100,001	136,222	72,412	301,639	11.85
>10000	fail	1,350,427,678	13,250	1,888,759	101,919.07	100,013	5,123	75,157	11,389	5.48
>20000	all	36,469,845,041	365,634	1,888,759	99,744.13	100,001	141,345	72,536	313,009	11.62
>20000	pass	35,119,417,363	352,384	670,868	99,662.35	100,001	136,222	72,412	301,639	11.85
>20000	fail	1,350,427,678	13,250	1,888,759	101,919.07	100,013	5,123	75,157	11,389	5.48
>30000	all	36,469,845,041	365,634	1,888,759	99,744.13	100,001	141,345	72,536	313,009	11.62
>30000	pass	35,119,417,363	352,384	670,868	99,662.35	100,001	136,222	72,412	301,639	11.85
>30000	fail	1,350,427,678	13,250	1,888,759	101,919.07	100,013	5,123	75,157	11,389	5.48
>40000	all	36,469,845,041	365,634	1,888,759	99,744.13	100,001	141,345	72,536	313,009	11.62
>40000	pass	35,119,417,363	352,384	670,868	99,662.35	100,001	136,222	72,412	301,639	11.85
>40000	fail	1,350,427,678	13,250	1,888,759	101,919.07	100,013	5,123	75,157	11,389	5.48
>50000	all	36,469,845,041	365,634	1,888,759	99,744.13	100,001	141,345	72,536	313,009	11.62
>50000	pass	35,119,417,363	352,384	670,868	99,662.35	100,001	136,222	72,412	301,639	11.85
>50000	fail	1,350,427,678	13,250	1,888,759	101,919.07	100,013	5,123	75,157	11,389	5.48
>60000	all	36,469,845,041	365,634	1,888,759	99,744.13	100,001	141,345	72,536	313,009	11.62
>60000	pass	35,119,417,363	352,384	670,868	99,662.35	100,001	136,222	72,412	301,639	11.85
>60000	fail	1,350,427,678	13,250	1,888,759	101,919.07	100,013	5,123	75,157	11,389	5.48
>70000	all	34,298,027,450	333,709	1,888,759	102,778.25	102,387	130,613	75,914	286,669	11.60
>70000	pass	32,952,493,367	320,529	670,868	102,806.59	102,478	125,519	75,940	275,341	11.85
>70000	fail	1,345,534,083	13,180	1,888,759	102,089.08	100,140	5,099	75,326	11,330	5.48

>80000	all	28,526,804,847	256,627	1,888,759	111,160.57	109,412	103,334	85,170	222,067	11.60
>80000	pass	27,432,842,084	246,804	670,868	111,152.34	109,464	99,447	85,191	213,574	11.84
>80000	fail	1,093,962,763	9,823	1,888,759	111,367.48	108,081	3,889	84,676	8,494	5.48
>90000	all	23,115,882,700	192,842	1,888,759	119,869.54	117,141	79,417	94,546	167,778	11.59
>90000	pass	22,248,192,536	185,688	670,868	119,814.92	117,156	76,537	94,552	161,566	11.83
>90000	fail	867,690,164	7,154	1,888,759	121,287.41	116,726	2,880	94,368	6,212	5.48
>100000	all	18,235,052,686	141,346	1,888,759	129,010.04	125,455	59,265	104,103	123,470	11.58
>100000	pass	17,559,830,479	136,223	670,868	128,905.03	125,440	57,181	104,104	119,009	11.81
>100000	fail	675,222,207	5,123	1,888,759	131,802.11	126,052	2,085	104,080	4,462	5.47
>110000	all	14,041,709,502	101,313	1,888,759	138,597.31	134,332	43,100	113,900	88,765	11.56
>110000	pass	13,521,260,839	97,668	670,868	138,441.05	134,295	41,609	113,904	85,586	11.78
>110000	fail	520,448,663	3,645	1,888,759	142,784.27	135,616	1,492	113,749	3,180	5.46
>120000	all	10,662,090,604	71,859	1,888,759	148,375.16	143,573	30,916	123,693	63,106	11.53
>120000	pass	10,266,459,966	69,302	670,868	148,140.89	143,520	29,870	123,680	60,874	11.75
>120000	fail	395,630,638	2,557	1,888,759	154,724.54	145,374	1,047	124,009	2,233	5.46
>130000	all	7,998,288,229	50,498	1,888,759	158,388.22	152,958	21,919	133,504	44,426	11.51
>130000	pass	7,696,885,205	48,696	670,868	158,059.91	152,859	21,187	133,489	42,853	11.73
>130000	fail	301,403,024	1,802	1,888,759	167,260.28	155,682	733	133,995	1,574	5.45
>140000	all	5,935,213,109	35,178	1,888,759	168,719.46	162,543	15,365	143,509	30,990	11.48
>140000	pass	5,705,651,377	33,909	670,868	168,263.63	162,360	14,856	143,508	29,883	11.70
>140000	fail	229,561,732	1,269	1,888,759	180,899.71	167,240	510	143,531	1,108	5.42
>150000	all	4,389,830,179	24,498	1,888,759	179,191.37	172,697	10,747	153,311	21,604	11.43
>150000	pass	4,213,902,774	23,600	670,868	178,555.2	172,555	10,394	153,288	20,821	11.66
>150000	fail	175,927,405	898	1,888,759	195,910.25	178,566	355	153,942	783	5.36
>160000	all	3,204,062,894	16,831	1,888,759	190,366.76	182,920	7,406	163,439	14,850	11.35
>160000	pass	3,068,005,833	16,191	670,868	189,488.35	182,673	7,161	163,402	14,294	11.59

>160000	fail	136,057,061	640	1,888,759	212,589.16	189,358	246	164,536	557	5.33
>170000	all	2,382,477,688	11,841	1,888,759	201,205.78	193,329	5,218	173,427	10,454	11.29
>170000	pass	2,276,113,444	11,381	670,868	199,992.39	193,090	5,049	173,400	10,056	11.53
>170000	fail	106,364,244	460	1,888,759	231,226.62	205,534	170	174,279	399	5.28
>180000	all	1,752,150,839	8,233	1,888,759	212,820.46	204,272	3,629	183,398	7,269	11.21
>180000	pass	1,668,883,691	7,905	670,868	211,117.48	203,653	3,516	183,355	6,987	11.46
>180000	fail	83,267,148	328	1,888,759	253,863.26	225,126	116	184,715	283	5.19
>190000	all	1,306,580,938	5,819	1,888,759	224,537.02	215,627	2,565	193,818	5,139	11.11
>190000	pass	1,239,568,151	5,579	670,868	222,184.65	214,836	2,488	193,778	4,933	11.37
>190000	fail	67,012,787	240	1,888,759	279,219.95	252,644	82	195,072	206	5.05
>200000	all	984,718,827	4,166	1,888,759	236,370.34	226,814	1,836	203,751	3,679	11.02
>200000	pass	927,010,351	3,974	670,868	233,268.84	225,713	1,777	203,657	3,515	11.31
>200000	fail	57,708,476	192	1,888,759	300,564.98	281,023	64	206,293	164	4.93

Table S3. Statistics of PacBio HiFi sequencing data.

Parameter	Value
HiFi reads	1,934,889
Total bases (Gb)	36.14
HiFi read length (bp)	18,676
HiFi read length N50 (bp)	19,410

Table S4. Statistics of next-generation sequencing data.

Parameter	Raw data	Clean data
Number of reads	650,939,538	650,939,534
Number of bases	97,640,930,700	95,848,003,498
GC content (%)	35.56	35.27
Q20 (%)	98.07	98.07
Q30 (%)	94.16	94.16

Table S5. Statistics of Hi-C sequencing data.

Parameter	Value
Raw reads	441,798,292
Raw bases	66,269,743,800
Clean reads	441,798,290
Clean bases	65,906,544,032
Q20 (%)	95.621
Q30 (%)	87.662

Table S6. Statistics of mixed preliminary assembly results by ONT and HiFi.

Parameter	Value
Length (bp)	432,597,900
Number of scaffolds	11
GC content (%)	34.89
N50 (bp)	45,693,234
N90 (bp)	41,673,157
Scaffold average length (bp)	39,327,082
Scaffold median length (bp)	45,128,179
Scaffold minimum length (bp)	1,035,235
Scaffold maximum length (bp)	57,471,326

Table S7. Statistics of genome consistency.

Parameter	Value
Mapping rate (%)	99.74
Average sequencing depth	205.46
Coverage (%)	99.97
Coverage at least 4× (%)	99.9
Coverage at least 10× (%)	99.8
Coverage at least 20× (%)	99.64

Table S8. Statistics of BUSCO evaluation.

Parameter	Value	Percent (%)
Complete BUSCOs (C)	1,597	98.9
Complete and single-copy BUSCOs (S)	1,529	94.7
Complete and duplicated BUSCOs (D)	68	4.2
Fragmented BUSCOs (F)	8	0.5
Missing BUSCOs (M)	9	0.6
Total BUSCO group searched	1,614	100.0

Table S9. Statistics of QV values of each chromosome in the genome.

ChrID	QV
chr1	51.37
chr2	55.29
chr3	55.07
chr4	48.84
chr5	51.54
chr6	57.55
chr7	59.15
chr8	52.54
chr9	59.53

Table S10. Basic statistics of repeated sequences in *D. carota* vT2T genome.

Type	TE proteins		*de novo* + RepBase		Combined TEs	
	Length (bp)	% in genome	Length (bp)	% in genome	Length (bp)	% in genome
DNA	2,998,784	0.70	12,394,840	2.88	12,942,187	3.01
LINE	2,396,109	0.56	4,568,174	1.06	5,148,317	1.20
SINE	0	0.00	6,700	0.00	6,700	0.00
LTR	32,588,301	7.57	129,536,132	30.10	130,392,028	30.30
LTR-Gypsy	10,090,734	2.34	32,173,458	7.48	32,839,942	7.63
LTR-Copia	22,127,770	5.14	77,892,800	18.10	78,404,056	18.22
Satellite	0	0.00	307,382	0.07	307,382	0.07
Simple_repeat	0	0.00	22,395	0.01	22,395	0.01
Other	294	0.00	1,313	0.00	1,607	0.00
Unknown	21,309	0.00	95,126,118	22.10	95,147,427	22.11
Total	37,990,298	8.83	231,748,596	53.85	238,008,817	55.30

Note: DNA, DNA transposons; LINE, long interspersed nuclear element; SINE, short interspersed element; LTR, long terminal repeat; Satellite, satellite DNA; Simple_repeat, simple sequence repeat; Other, unclassified DNA transposons; Unknown, unclassified repeats that can be labeled.

Table S11. Basic statistics of gene structure prediction in *D. carota* vT2T genome.

Method		Gene number	Average gene length (bp)	Average CDS length (bp)	Average exon per gene	Average exon length (bp)	Average intron length (bp)
Ab initio	GlimmmerHMM	57,412	6,759.87	1,070.48	4.20	254.86	1,777.76
	AUGUSTUS	46,193	3,555.70	1,195.65	5.34	223.91	543.82
Homology-based	*Apium graveolens*	51,965	6,783.31	789.68	3.45	229.11	2,449.62
	Coriandrum sativum	61,196	7,465.71	780.37	3.28	237.70	2,928.28
	Angelica sinensis	61,983	7,713.75	770.73	3.18	242.42	3,185.89
	Oenanthe sinensis	68,713	12,007.77	847.61	3.38	250.84	4,690.89
RNAseq		21,664	5,329.78	1,227.42	6.49	317.65	595.73
Integration		35,402	4,972.18	1,252.42	5.66	277.27	729.93
Final set		36,268	4,630.72	1,202.11	5.38	301.98	684.09

Table S12. Statistic of functional annotation of coding genes in *D. carota* vT2T genome.

Database	Count	Per centage (%)
Annotation	34,961	96.40
KEGG	8,874	24.47
Pathway	6,976	19.23
Nr	34,562	95.30
Uniprot	34,132	94.11
GO	24,189	66.70
KOG	202	0.56
Pfam	24,468	67.46
InterPro	33,238	91.65

Table S13. Statistic of non-coding RNA annotation results in *D. carota* vT2T genome.

Type		Copy	Average length (bp)	Total length (bp)	% of genome
miRNA		123	127	15,599	0.003624
tRNA		500	77	38,263	0.008890
rRNA	rRNA	1,598	122	194,786	0.045257
	18S	3	870	2,610	0.000606
	28S	4	200	802	0.000186
	5.8S	4	166	663	0.000154
	5S	1,587	120	190,711	0.044310
snRNA	snRNA	772	119	91,601	0.021283
	CD-box	582	110	63,805	0.014825
	HACA-box	36	127	4,565	0.001061
	splicing	154	151	23,231	0.005398
	scaRNA	0	0	0	0.000000

Table S14. Carotenoid pathway gene mining in *D. carota* vT2T genome.

Gene	Gene number	Location	CDS Length	Number in *Daucus carota* v2.0 genome	Pfam database annotation
PSY	DcarChr3G00111890.1	chr3:14,453,271:14,458,167	1,317	DCAR_010057	PF00494.22//SQS_PSY`Squalene/phytoene synthase
	DcarChr6G00218740.1	chr6:3,389,587:3,391,853	1,197	DCAR_023043	PF00494.22//SQS_PSY`Squalene/phytoene synthase
	DcarChr7G00301710.1	chr7:15,351,089:15,353,311	1,158	DCAR_024333	PF00494.22//SQS_PSY`Squalene/phytoene synthase
PDS	DcarChr5G00173560.1	chr5:194,285:203,724	1,977	DCAR_016085	PF13450.9//NAD_binding_8`NAD(P)-binding Rossmann-like domain; PF01593.27//Amino_oxidase`Flavin containing amine oxidoreductase
ZDS	DcarChr7G00312090.1	chr7:32,516,698:32,527,011	1,728	DCAR_025321	PF13450.9//NAD_binding_8`NAD(P)-binding Rossmann-like domain; PF01593.27//Amino_oxidase`Flavin containing amine oxidoreductase
	DcarChr2G00074060.1	chr2:40,791,155:40,797,679	1,722	DCAR_006616	PF13450.9//NAD_binding_8`NAD(P)-binding Rossmann-like domain; PF01593.27//Amino_oxidase`Flavin containing amine oxidoreductase
CRTISO	DcarChr1G00014980.1	chr1:25,778,714:25,790,715	1,740	DCAR_001700	PF13450.9//NAD_binding_8`NAD(P)-binding Rossmann-like domain; PF01593.27//Amino_oxidase`Flavin containing amine oxidoreductase
	DcarChr5G00187770.1	chr5:14,082,445:14,099,440	1,677	DCAR_017290	PF13450.9//NAD_binding_8`NAD(P)-binding Rossmann-like domain; PF01593.27//Amino_oxidase`Flavin containing amine

					oxidoreductase
Z-ISO	DcarChr7G00294210.1	chr7:5,502,049:5,504,467	1,098	DCAR_023800	PF07298.14//NnrU`NnrU protein
LCYB	DcarChr6G00247320.1	chr6:39,041,891:39,044,224	1,527	DCAR_020544	PF05834.15//Lycopene_cycl`Lycopene cyclase protein
	DcarChr6G00220470.1	chr6:5,184,455:5,185,933	1,479	DCAR_022896	PF05834.15//Lycopene_cycl`Lycopene cyclase protein
LCYE	DcarChr8G00149410.1	chr8:21,153,595:21,159,070	1,593	DCAR_028276	PF05834.15//Lycopene_cycl`Lycopene cyclase protein
BCH	DcarChr6G00250300.1	chr6:41,372,381:41,375,200	930	DCAR_020269	PF04116.16//FA_hydroxylase`Fatty acid hydroxylase
	DcarChr4G00343080.1	chr4:24,790,508:24,792,526	912	DCAR_014519	PF04116.16//FA_hydroxylase`Fatty acid hydroxylase
	DcarChr3G00104490.1	chr3:7,325,122:7,333,157	684	DCAR_009395	PF04116.16//FA_hydroxylase`Fatty acid hydroxylase
CYP97C1/lut1	DcarChr5G00192600.1	chr5:25,466,146:25,474,510	1,620	DCAR_017658	PF00067.25//p450`Cytochrome P450
	DcarChr4G00355680.1	chr4:34,477,145:34,483,312	1,758	DCAR_013342	PF00067.25//p450`Cytochrome P450
	DcarChr5G00182230.1	chr5:8,152,562:8,153,989	1,428	-	PF00067.25//p450`Cytochrome P450
	DcarChr8G00168330.1	chr8:42,037,621:42,039,609	1,536	DCAR_026858	PF00067.25//p450`Cytochrome P450
	DcarChr2G00077020.1	chr2:43,014,198:43,016,137	1,515	DCAR_006881	PF00067.25//p450`Cytochrome P450
	DcarChr5G00182280.1	chr5:8,171,139:8,172,674	1,536	-	PF00067.25//p450`Cytochrome P450
	DcarChr8G00170540.1	chr8:43,720,546:43,722,859	1,545	DCAR_026686	PF00067.25//p450`Cytochrome P450
	DcarChr9G00272910.1	chr9:29,096,550:29,100,517	1,773	-	PF00067.25//p450`Cytochrome P450
	DcarChr4G00356300.1	chr4:34,931,945:34,936,154	1,557	DCAR_013285<br>DCAR_013284	PF00067.25//p450`Cytochrome P450
	DcarChr5G00182260.1	chr5:8,163,191:8,164,702	1,512	-	PF00067.25//p450`Cytochrome P450
	DcarChr4G00342040.1	chr4:24,054,964:24,062,156	1,581	DCAR_014607<br>DCAR_014606	PF00067.25//p450`Cytochrome P450
	DcarChr5G00183190.1	chr5:8,989,960:8,992,438	1,560	DCAR_016896	PF00067.25//p450`Cytochrome P450

	DcarChr4G00351650.1	chr4:31,610,393:31,615,718	1,545	-	PF00067.25//p450`Cytochrome P450
	DcarChr5G00191650.1	chr5:24,304,524:24,307,321	1,617	DCAR_017575<br>DCAR_017576	PF00067.25//p450`Cytochrome P450
	DcarChr9G00261390.1	chr9:5,794,134:5,796,441	1,551	-	PF00067.25//p450`Cytochrome P450
	DcarChr4G00356690.1	chr4:35,235,954:35,238,549	1,569	-	PF00067.25//p450`Cytochrome P450
CYP97A3/lut5	DcarChr8G00168340.1	chr8:42,040,527:42,042,548	1,635	DCAR_026857	PF00067.25//p450`Cytochrome P450
	DcarChr7G00294670.1	chr7:6,019,723:6,029,683	1,755	DCAR_023843	PF00067.25//p450`Cytochrome P450
	DcarChr5G00182290.1	chr5:8,174,296:8,178,119	2,382	-	PF00067.25//p450`Cytochrome P450
	DcarChr8G00162410.1	chr8:37,118,269:37,120,636	1,602	DCAR_027427	PF00067.25//p450`Cytochrome P450
	DcarChr6G00250480.1	chr6:41,489,925:41,492,445	1,572	DCAR_020252<br>DCAR_020253	PF00067.25//p450`Cytochrome P450
	DcarChr3G00126310.1	chr3:37,840,325:37,841,860	1,536	DCAR_014040<br>DCAR_014011	PF00067.25//p450`Cytochrome P450
	DcarChr4G00341500.1	chr4:23,603,546:23,606,662	1,650	DCAR_014655	PF00067.25//p450`Cytochrome P450
	DcarChr1G00025750.1	chr1:37,659,119:37,661,102	1,410	DCAR_002585	PF00067.25//p450`Cytochrome P450
ZEP	DcarChr7G00316060.1	chr7:35,672,400:35,678,747	2,007	DCAR_025735	PF01494.22//FAD_binding_3`FAD binding domain;<br>PF00498.29//FHA`FHA domain
	DcarChr4G00328470.1	chr4:12,648,677:12,651,611	1,422	DCAR_015695	PF01494.22//FAD_binding_3`FAD binding domain
	DcarChr1G00001630.1	chr1:1,753,286:1,756,937	966	DCAR_000648	PF01494.22//FAD_binding_3`FAD binding domain;<br>PF08491.13//SE`Squalene epoxidase
	DcarChr3G00105780.1	chr3:8,475,024:8,477,930	1,281	DCAR_009506<br>DCAR_009507	PF01494.22//FAD_binding_3`FAD binding domain
	DcarChr1G00011800.1	chr1:21,314,676:21,316,822	1,272	DCAR_001412<br>DCAR_001413	PF01494.22//FAD_binding_3`FAD binding domain;<br>PF13450.9//NAD_binding_8`NAD(P)-binding Rossmann-like domain
	DcarChr4G00348040.1	chr4:28,889,654:28,894,097	1,188	DCAR_014050	PF01494.22//FAD_binding_3`FAD binding domain;

					PF13450.9//NAD_binding_8`NAD(P)-binding Rossmann-like domain
NCED	DcarChr2G00050160.1	chr2:568,086:569,870	1,785	DCAR_004823	PF03055.18//RPE65`Retinal pigment epithelial membrane protein
	DcarChr4G00350790.1	chr4:31,008,287:31,010,053	1,767	DCAR_013799	PF03055.18//RPE65`Retinal pigment epithelial membrane protein
	DcarChr5G00191550.1	chr5:24,093,840:24,095,606	1,767	DCAR_017563	PF03055.18//RPE65`Retinal pigment epithelial membrane protein
	DcarChr5G00214070.1	chr5:44,545,281:44,546,996	1,716	DCAR_019639	PF03055.18//RPE65`Retinal pigment epithelial membrane protein
	DcarChr6G00241930.1	chr6:34,970,650:34,972,389	1,740	DCAR_021059	PF03055.18//RPE65`Retinal pigment epithelial membrane protein
	DcarChr8G00156260.1	chr8:30,864,360:30,866,102	1,743	DCAR_027098	PF03055.18//RPE65`Retinal pigment epithelial membrane protein
	DcarChr1G00022330.1	chr1:34,673,307:34,674,803	1,497	DCAR_002269	PF03055.18//RPE65`Retinal pigment epithelial membrane protein
CCD	DcarChr6G00226530.1	chr6:21,997,669:22,007,180	1,644	DCAR_022385	PF03055.18//RPE65`Retinal pigment epithelial membrane protein
	DcarChr9G00284360.1	chr9:41,670,939:41,673,283	1,767	DCAR_030904	PF03055.18//RPE65`Retinal pigment epithelial membrane protein
	DcarChr1G00032650.1	chr1:43,694,374:43,702,273	1,323	DCAR_003216	PF03055.18//RPE65`Retinal pigment epithelial membrane protein
	DcarChr6G00226490.1	chr6:21,974,335:21,975,864	558	DCAR_022390	PF03055.18//RPE65`Retinal pigment epithelial membrane protein
	DcarChr1G00031650.1	chr1:42,758,437:42,766,019	1,845	DCAR_003120	PF03055.18//RPE65`Retinal pigment epithelial membrane protein

	DcarChr1G00031690.1	chr1:42,803,362:42,810,120	1,836	DCAR_003121	PF03055.18//RPE65`Retinal pigment epithelial membrane protein
	DcarChr6G00226480.1	chr6:21,971,692:21,974,295	732	DCAR_022390	PF03055.18//RPE65`Retinal pigment epithelial membrane protein
	DcarChr1G00046240.1	chr1:54,541,793:54,544,919	1,689	DCAR_004470	PF03055.18//RPE65`Retinal pigment epithelial membrane protein
	DcarChr1G00032640.1	chr1:43,689,052:43,689,665	168	DCAR_003216	PF03055.18//RPE65`Retinal pigment epithelial membrane protein
	DcarChr9G00267770.1	chr9:14,918,692:14,922,842	1,869	DCAR_031889	PF03055.18//RPE65`Retinal pigment epithelial membrane protein
	DcarChr7G00317930.1	chr7:37,209,186:37,211,363	726	DCAR_025914	PF14108.9//ABA4-like`ABA DEFICIENT 4-like
NXS	DcarChr4G00330350.1	chr4:14,263,386:14,266,178	726	-	PF14108.9//ABA4-like`ABA DEFICIENT 4-like
	DcarChr5G00186580.1	chr5:12,540,690:12,543,200	744	DCAR_017191	PF14108.9//ABA4-like`ABA DEFICIENT 4-like

Table S15. Transposons overlapped with carotenoid pathway genes in *D. carota* genome.

Gene Number	Transposon location	Target	Class	PercDiv	PercDel	PercIns
DcarChr7G00301710.1 (*PSY*)	15,351,865-15,351,918	rnd-4_family-683	Unknown	20.4	1.9	0.0
DcarChr5G00173560.1 (*PDS*)	194,561-194,681	rnd-4_family-51	LTR/Copia	25.8	9.1	0.8
	197,400-197,716	DF0001793	Satellite	10.9	0.0	0.3
	197,666-197,751	Tx1-11_CGi	LINE/L1-Tx1	21.2	1.2	1.2
	198,885-198,909	DF0003501	DNA/Kolobok-T2	23.6	11.6	1.4
	198,910-198,934	EnSpm-N4_HM	DNA/CMC-EnSpm	19.0	1.0	4.0
	198,935-199,012	RTEX-3_SSa	LINE/RTE-X	15.6	1.3	1.3
	198,960-199,061	rnd-1_family-0	Unknown	20.0	2.9	1.9
	198,983-199,404	chr2: 21699761..21702249_INT	LTR/unknown	25.5	7.0	6.5
	199,335-199,414	rnd-6_family-10317	Unknown	25.0	6.2	0.0
	199,359-199,420	rnd-1_family-0	Unknown	16.7	0.0	3.3
	199,410-199,810	rnd-1_family-55	Unknown	6.7	0.2	1.7
	199,811-199,853	EnSpm-12_OS	DNA/CMC-EnSpm	19.1	7.5	2.1
	199,854-199,940	chr4: 1272390..1274308_LTR	LTR/unknown	17.2	6.9	0.0
	199,872-199,954	rnd-5_family-2214	Unknown	16.9	3.6	0.0
	199,955-200,398	rnd-1_family-55	Unknown	6.7	0.2	1.7
	200,001-200,398	rnd-1_family-45	Unknown	10.2	0.0	3.9
	200,359-200,369	Kolobok-N1_AEc	DNA/Kolobok-T2	22.0	0.0	1.7
	200,370-200,413	Zisupton-6_DR	DNA/Zisupton	11.4	0.0	0.0
	200,376-200,426	ERV1-4_AFC-int	LTR/ERV1	21.6	2.0	0.0
DcarChr5G00187770.1 (*CRTISO*)	14,082,969-14,083,205	rnd-1_family-9	Unknown	15.6	0.4	0.0
	14,083,547-14,083,627	rnd-1_family-675	Unknown	28.4	0.0	0.0
	14,083,763-14,083,817	rnd-1_family-675	Unknown	23.6	0.0	0.0

	14,085,438-14,085,555	rnd-6_family-5557	Unknown	13.4	3.4	5.2
	14,085,785-14,086,128	rnd-6_family-5557	Unknown	6.8	5.5	1.7
	14,089,098-14,089,156	Copia-4_PAb-I	LTR/Copia	25.4	5.1	0.0
	14,089,879-14,089,979	chr8: 34787707..34796261_INT	LTR/Copia	15.8	4.0	6.1
	14,089,906-14,090,111	chr3: 6167792..6172795_INT	LTR/unknown	6.7	0.5	5.6
	14,089,946-14,090,133	chr9: 3671594..3686047_INT	LTR/unknown	14.2	5.8	6.4
	14,090,089-14,090,375	chr3: 6167792..6172795_INT	LTR/unknown	13.2	4.5	4.9
	14,090,120-14,090,529	rnd-6_family-1992	Unknown	4.4	8.2	0.4
	14,092,505-14,092,781	chr5: 14092505..14092781_LTR	LTR/Gypsy	0.0	0.0	0.0
	14,092,782-14,097,404	chr5: 14092782..14097404_INT	LTR/Gypsy	0.0	0.0	0.0.
	14,097,405-14,097,681	chr5: 14092505..14092781_LTR	LTR/Gypsy	0.0	0.0	00
	14,097,682-14,098,120	rnd-5_family-10802	Unknown	1.8	0.2	0.4
DcarChr8G00149410.1 (*LCYE*)	21,153,889-21,154,224	chr6: 11390356..11397777_INT	LTR/Copia	8.6	0.0	0.0
	21,154,225-21,157,216	rnd-5_family-130	Unknown	12.9	6.1	1.8
	21,157,217-21,157,546	chr6: 11390356..11397777_INT	LTR/Copia	13.3	0.0	0.0
	21,157,547-21,158,392	rnd-5_family-130	Unknown	12.9	6.1	1.8
	21,158,393-21,158,722	chr6: 11390356..11397777_INT	LTR/Copia	13.0	0.0	0.0
	21,158,723-21,160,125	rnd-5_family-130	Unknown	12.9	6.1	1.8
DcarChr4G00343080.1 (*BCH*)	24,791,214-24,791,273	rnd-1_family-706	Unknown	13.3	1.7	0.0
	24,791,232-24,791,420	rnd-4_family-760	Unknown	7.5	2.1	7.8
DcarChr4G00355680.1 (*CYP97C1*)	34,477,416-34,477,830	rnd-5_family-1116	DNA/MULE-MuDR	30.2	4.0	3.4
	34,478,002-34,478,682	MuDR-110_OS	DNA/MULE-MuDR	31.5	4.1	3.6
	34,479,389-34,479,614	rnd-5_family-3885	Unknown	22.0	4.0	1.3
	34,479,534-34,479,675	rnd-1_family-0	Unknown	15.2	4.5	3.0
	34,479,568-34,479,689	DF0003162	DNA/MULE-MuDR	12.1	3.3	5.0
	34,479,595-34,479,701	DF0003936	LTR/Ngaro	16.3	3.7	2.8

	34,479,617-34,479,706	DF0003505	DNA/hAT-Tip100	7.8	3.3	0.0
	34,479,707-34,479,749	Sola2-1_HM	DNA/Sola-2	12.2	8.3	1.4
	34,479,750-34,479,773	EnSpm-N10_HM	DNA/CMC-EnSpm	14.4	7.6	3.0
	34,480,091-34,480,243	rnd-6_family-1565	Unknown	15.3	5.9	1.9
	34,480,239-34,480,730	chr4: 2659194..2661607_INT	LTR/unknown	14.7	8.1	2.9
	34,480,775-34,480,916	rnd-3_family-209	Unknown	22.8	0.7	13.5
	34,480,909-34,481,324	rnd-1_family-436	Unknown	13.3	0.5	1.0
	34,481,340-34,481,517	rnd-6_family-7639	Unknown	17.9	2.3	3.4
	34,481,640-34,482,033	rnd-5_family-1329	LTR/Copia	9.5	0.0	1.6
	34,482,022-34,482,151	chr2: 35382426..35395665_INT	LTR/Copia	22.7	2.3	1.5
	34,482,032-34,482,244	chr4: 36545742..36551061_INT	LTR/unknown	25.7	5.2	5.2
	34,482,677-34,482,702	chr3: 21786148..21794447_INT	LTR/Gypsy	25.2	3.0	3.6
	34,482,703-34,482,845	chr1: 723206..730236_INT	LTR/Copia	26.9	2.1	1.4
	34,482,847-34,482,913	rnd-5_family-1329	LTR/Copia	13.4	0.0	0.0
DcarChr8G00170540.1 (*CYP97C1*)	43,722,253-43,722,599	rnd-5_family-567	Unknown	29.2	2.9	2.3
DcarChr5G00182280.1 (*CYP97C1*)	8,171,173-8,172,705	rnd-6_family-17868	Unknown	18.7	1.0	0.2
DcarChr5G00182260.1 (*CYP97C1*)	8,163,191-8,164,697	rnd-6_family-17868	Unknown	17.4	2.3	0.3
DcarChr9G00272910.1 (*CYP97C1*)	29,096,545-29,096,601	rnd-6_family-1	Unknown	12.3	1.8	0.0
	29,097,349-29,097,548	rnd-6_family-505	Unknown	10.6	1.0	1.0
	29,098,102-29,098,439	chr5: 31498843..31503303_INT	LTR/Copia	32.4	2.1	2.4
	29,098,166-29,098,473	Copia1-PTR_I	LTR/Copia	34.4	1.6	1.9
	29,098,416-29,098,653	rnd-5_family-1836	LTR/Copia	25.1	1.1	1.9
	29,098,829-29,098,978	rnd-6_family-17868	Unknown	16.7	8.7	0.0

	29,098,981-29,100,542	rnd-6_family-17868	Unknown	14.1	0.2	0.3
DcarChr4G00351650.1 (*CYP97C1*)	31,610,390-31,611,931	rnd-6_family-17868	Unknown	18.7	0.6	1.0
	31,612,258-31,612,302	Gypsy-73_OS-I	LTR/Gypsy	15.6	4.4	0.0
	31,613,320-31,613,530	rnd-1_family-657	Unknown	10.3	2.8	3.8
	31,614,210-31,615,811	rnd-6_family-17868	Unknown	17.6	1.2	0.9
DcarChr3G00126310.1 (*CYP97A3*)	37,840,348-37,840,936	rnd-6_family-17868	Unknown	30.4	1.2	0.7
DcarChr9G00284360.1 (*CCD*)	41,670,857-41,671,032	rnd-3_family-209	Unknown	21.1	24.4	0.5
DcarChr1G00046240.1 (*CCD*)	54,542,566-54,542,634	rnd-6_family-4587	Unknown	16.4	1.4	2.9
	54,542,644-54,542,740	rnd-1_family-172	Unknown	10.5	4.6	0.0
DcarChr1G00032640.1 (*CCD*)	43,689,215-43,689,268	BEL-247_AA-I	LTR/Pao	16.1	0.0	8.0
DcarChr6G00226480.1 (*CCD*)	21,972,817-21,973,043	EnSpm-13_ZM	DNA/CMC-EnSpm	24.8	2.6	5.9
DcarChr4G00330350.1 (*NXS*)	14,265,906-14,265,980	rnd-6_family-505	Unknown	17.6	0.0	1.4
DcarChr7G00317930.1 (*NXS*)	37,209,464-37,209,578	rnd-6_family-505	Unknown	14.3	0.1	0.4
	37,209,464-37,209,578	rnd-6_family-505	Unknown	14.3	0.1	0.4
	37,211,084-37,211,161	rnd-6_family-505	Unknown	14.5	0.0	2.6

Note: PercDiv, per centage of divergence; PercDel, per centage of delete; PercIns, per centage of insert

Table S16. Primers used for gene expression analysis.

Gene	Primer-Forward (5'→3')	Primer-Reverse (5'→3')
DcarChr6G00218740.1	GAATGAGGCCTACGATCGGTGT	CTTGGGCGTGATATGGGAGG
DcarChr3G00111890.1	TGTCAGTTGCTATGTCCTGGATTGT	CTTACCCTTCTCAAGTCTGCCTCC
DcarChr5G00173560.1	AAGTCAAGTTTGCGTTGGGTCTC	CGAAAACCTCAGTTGTAACCCGA
DcarChr7G00294210.1	GTTTGGAATGGTGACAGAAGGCT	CCTCGAGAATAGCTGCAAATGGTA
DcarChr7G00312090.1	TATCTATTTTCCGGCCACTTCC	GCTTTGGTCCACGATAATGTTCTG
DcarChr2G00074060.1	GAGGATGATGATGCTTGTTCGC	CCGCAAGCCCAGCTCCTATAA
DcarChr5G00187770.1	GCATCTTTGAAACCCCATTACCA	GCTTCCTCTTTCTCCTACTCACCC
DcarChr6G00247320.1	TTGACCTTCCTTTGTATGACCCG	CTGCCTCAGAAACTTGTTGTGCT
DcarChr6G00220470.1	GCGAGGCTCAGACATTTAGGAA	TGCCACCATGTACCCTGTTGAA
DcarChr8G00149410.1	GTGGCAGGGATTTCTTGGTTCT	TTACAAGGGACATTCTCAAGTGGTG
DcarChr5G00192600.1	AAGCCCAAGCAGAAGTTGATAGAGT	CTCATTTATGCAGCGTGTCAGGTA
DcarChr4G00356690.1	ACCAACCAAGAGTAACAAGAGG	CACATCGTCCAGGCTCATTC
DcarChr8G00170540.1	GACACAGATTGGCAGGATA	TGATGAAGAGCGAGTATGG
DcarChr2G00077020.1	GCTTGCTAGTGTTGAGTTCTCC	GCCAATGTTAAGAAGTCGCTGTA
DcarChr4G00355680.1	ATGACCGCCAACTAAGAGATGA	GCAGAACCTCCACAACAATAAGA
DcarChr4G00342040.1	CTTGTGTTCTGGAGATATGCTGAT	TCTTGCGGTGGATGAGGTC
DcarChr6G00250300.1	GCGAGGAAGAAGTCGGAGAGGT	AAGAGAATCTGTAATAAACCGCCAA
DcarChr4G00343080.1	AGGTTCCTCACTCAGAGATGCTTG	ATCTTGCCCAGTACTCCATGCC
DcarChr3G00104490.1	CCTTGTGGCATACTTCATTG	AGCAGACGACTTCATACTTC
DcarChr7G00294670.1	CATTTCAATCTGGAACTTACATCGC	GGTCTCATTTGGGTTGGGTCC
DcarChr4G00341500.1	CGTGGTGCTTGATGCTTCTT	AATGCTGCTGGCGGATACA
DcarChr3G00126310.1	TCATCAGTTCCGCCGATTCT	CCGCTCACATCCTATATTCATCC
DcarChr5G00182290.1	CCTGGCTGAATGTGGACTCA	ATACAACCGCTGGCAGTGAT
DcarChr7G00317930.1	CAGTTCTCCTCTGCTTCTTA	TCCATTCGGTTCTACTCTTC

DcarChr5G00186580.1	AATATCTATTGCTGGTCCGACTATG	TGAATACACTTCTTGGTCAGAGTAG
DcarChr5G00214070.1	CTCTCCTCACTTGCCTTCTCACAT	GGGACTTGAATAGAAGAGTGCACAT
DcarChr4G00350790.1	TTGTGCTATACAATCATCGCCTTCT	GTACAATGGTAGTGGGAATCGAAGG
DcarChr5G00191550.1	GGACAAGTATGCGAAGGAT	AAGAGCCAATCACCACAAT
DcarChr1G00001630.1	CAGCAATCAAGTCCGAACCAA	TCCGAACAGAGCATACCAGAG
DcarChr7G00316060.1	TGGAGCGTGCTACAAAAGGAGA	GGTTTTCCTTCATCCTTGCTGAGA
DcarChr4G00348040.1	GTGGTTCAGCAGGCTTGGA	GCGAGGTAGATTCTGTTATGAGTAA
DcarChr1G00011800.1	GCATTGGCAACACTTATCTC	ACCGTGGAAGCATATTGAG
DcarChr9G00284360.1	AGACGGGGATTGTTTCTAGGGTT	GTTTTGGCCGACGTAAGATGG
DcarChr9G00267770.1	GTACATTAAAACCGACGCGAAAG	AACTCAAAACACTGGTGTTCGCC
DcarChr1G00046240.1	GAGACGCCTCCTGCTCGTGAT	CACATAGCGGGAGACATAGGTAGC
DcarChr6G00226530.1	CCGTCTGAGCCATTCTTCGTG	ATGATTTCCCATCTAAGACCAGTGC
